# Global analysis of protein-RNA interactions in SARS-CoV-2-infected cells reveals key regulators of infection

**DOI:** 10.1016/j.molcel.2021.05.023

**Published:** 2021-07-01

**Authors:** Wael Kamel, Marko Noerenberg, Berati Cerikan, Honglin Chen, Aino I. Järvelin, Mohamed Kammoun, Jeffrey Y. Lee, Ni Shuai, Manuel Garcia-Moreno, Anna Andrejeva, Michael J. Deery, Natasha Johnson, Christopher J. Neufeldt, Mirko Cortese, Michael L. Knight, Kathryn S. Lilley, Javier Martinez, Ilan Davis, Ralf Bartenschlager, Shabaz Mohammed, Alfredo Castello

**Affiliations:** 1 MRC-University of Glasgow Centre for Virus Research, G61 1QH Glasgow, Scotland, UK; 2 Department of Biochemistry, University of Oxford, South Parks Road, OX1 3QU Oxford, UK; 3 Department of Infectious Diseases, Molecular Virology, Heidelberg University, 69120 Heidelberg, Germany; 4 German Center for Infection Research, Heidelberg Partner Site, 69120 Heidelberg, Germany; 5 German Cancer Research Center (DKFZ), 69120 Heidelberg, Germany; 6 Sir William Dunn School of Pathology, University of Oxford, South Parks Road, OX1 3RE Oxford, UK; 7 Department of Biochemistry, University of Cambridge, CB2 1GA Cambridge, UK; 8 Center of Medical Biochemistry, Max Perutz Labs, Medical University of Vienna, Vienna, Austria; 9 Department of Chemistry, University of Oxford, Mansfield Road, OX1 3TA Oxford, UK; 10 Division Virus-Associated Carcinogenesis, Germany Cancer Research Center (DKFZ), 69120 Heidelberg, Germany; 11 The Rosalind Franklin Institute, OX11 0FA Oxfordshire, UK

**Keywords:** RNA, SARS-CoV-2, COVID-19, RNA-binding protein, RNA interactome, host-virus interactions, ribonucleoprotein, tRNA ligase, antivirals, viral replication, HSP90

## Abstract

Severe acute respiratory syndrome coronavirus 2 (SARS-CoV-2) causes coronavirus disease 2019 (COVID-19). SARS-CoV-2 relies on cellular RNA-binding proteins (RBPs) to replicate and spread, although which RBPs control its life cycle remains largely unknown. Here, we employ a multi-omic approach to identify systematically and comprehensively the cellular and viral RBPs that are involved in SARS-CoV-2 infection. We reveal that SARS-CoV-2 infection profoundly remodels the cellular RNA-bound proteome, which includes wide-ranging effects on RNA metabolic pathways, non-canonical RBPs, and antiviral factors. Moreover, we apply a new method to identify the proteins that directly interact with viral RNA, uncovering dozens of cellular RBPs and six viral proteins. Among them are several components of the tRNA ligase complex, which we show regulate SARS-CoV-2 infection. Furthermore, we discover that available drugs targeting host RBPs that interact with SARS-CoV-2 RNA inhibit infection. Collectively, our results uncover a new universe of host-virus interactions with potential for new antiviral therapies against COVID-19.

## Highlights

• A third of the RBPome remodels upon SARS-CoV-2 infection
• Viral RNPs include 139 cellular and 6 viral RBPs
• Inhibition of these cellular RBPs hampers SARS-CoV-2 infection
• The tRNA ligase complex is a key regulator of SARS-CoV-2

## Graphical abstract

**Figure:**
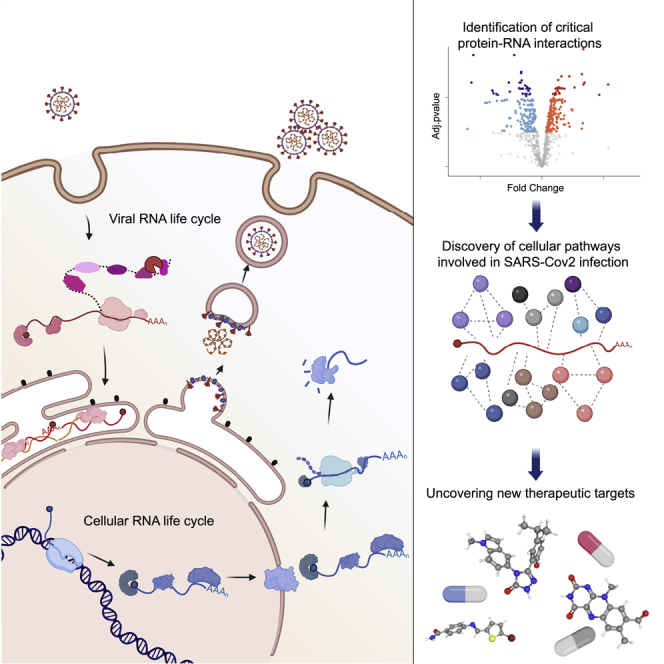

## Teaser

Kamel et al. apply a multi-omic approach to identify the RNA-binding proteins that regulate SARS-CoV-2 infection. They discovered that the complement of RNA-binding proteins heavily remodels upon SARS-CoV-2 infection. They also show that the viral RNA interacts with dozens of cellular and six viral RNA-binding proteins. These host-virus interactions are fundamental for SARS-CoV-2 infection and have great potential for new therapeutic approaches against COVID-19.

## Introduction

Severe acute respiratory syndrome coronavirus 2 (SARS-CoV-2) emerged in Wuhan, China, probably because of zoonotic transmission from bats (Zhou et al., 2020). It is the causative agent of coronavirus disease 2019 (COVID-19) and has become a pandemic (Dong et al., 2020). SARS-CoV-2 belongs to the *Coronaviridae* family and has a single-stranded, positive-sense RNA genome of ∼30 kb. It is an intracellular parasite that relies on host cell resources to replicate and spread. Hence, intensive efforts have been undertaken to improve our understanding of SARS-CoV-2 interactions with the host cell (Banerjee et al., 2020; Bojkova et al., 2020; Bouhaddou et al., 2020; Gordon et al., 2020; Kim et al., 2020b; Klann et al., 2020; Stukalov et al., 2020).

Most processes of the life cycle of RNA viruses are directed to multiply, transport, and deliver the viral RNA genome into a new cell. However, these viral genomes cannot encode all proteins required to accomplish these processes autonomously. To overcome this limitation, viruses hijack cellular RNA-binding proteins (RBPs) (Dicker et al., 2021; Garcia-Moreno et al., 2018). In response, the host cell employs specialized RBPs to detect viral RNAs and intermediates of replication through the recognition of unusual molecular signatures, including tri-phosphate ends, undermethylated cap, and double-stranded RNA (dsRNA) (Habjan and Pichlmair, 2015). RBP sensing of viral RNA triggers the cellular antiviral state, which can suppress viral gene expression through the inhibition of protein synthesis and the production of interferons. Therefore, cellular RBPs are key regulators of the virus life cycle, either promoting or restricting infection (Garcia-Moreno et al., 2018; Habjan and Pichlmair, 2015). It is thus fundamental to elucidate the interactions that SARS-CoV-2 RNA establishes with the host cell.

We recently developed comparative RNA interactome capture (cRIC) to discover how the RNA-bound proteome (RBPome) responds to Sindbis (SINV) infection (Garcia-Moreno et al., 2019). Our studies showed that SINV infection remodels the cellular RBPome and that these changes are critical for viral fitness (Garcia-Moreno et al., 2019). These observations highlight the essential role that RBPs play in regulating the viral life cycle (Dicker et al., 2021; Garcia-Moreno et al., 2018). In the last few years, several approaches have been developed to identify the cellular proteins that interact with viral RNA (Kim et al., 2020a; LaPointe et al., 2018; Ooi et al., 2019; Phillips et al., 2016; Viktorovskaya et al., 2016). Although these studies make important advances toward understanding viral ribonucleoproteins (RNPs), the choice of crosslinking and RNA isolation approaches may affect the results. For example, although formaldehyde is a more efficient crosslinker than ultraviolet (UV) light, it also promotes protein-protein crosslinks, allowing the capture of indirect interactions through protein-protein bridges (Tayri-Wilk et al., 2020). Despite their pros and cons, these studies discovered cellular proteins that engage with viral RNA in infected cells, revealing that the viral RNA is a hub for complex host-virus interactions (Kim et al., 2020a; Knoener et al., 2017; LaPointe et al., 2018; Ooi et al., 2019; Phillips et al., 2016; Schmidt et al., 2021; Viktorovskaya et al., 2016).

In this study, we employ multiple proteome-wide approaches to identify RBPs involved in the SARS-CoV-2 life cycle. We discover that the repertoire of cellular RBPs widely remodels in response to SARS-CoV-2 infection, affecting proteins involved in RNA metabolism, antiviral defenses, and other pathways. Moreover, we identify the cellular and viral proteins that interact with SARS-CoV-2 RNAs, employing a new approach named viral RNA interactome capture (vRIC). Dozens of cellular RBPs and six viral proteins are part of the SARS-CoV-2 RNPs, many of which lack known roles in virus infection. Furthermore, we show that pharmacological inhibition or dysregulation of cellular RBPs that interact with viral RNA impairs SARS-CoV-2 infection. Collectively, our data uncover the landscape of protein-RNA interactions that regulate SARS-CoV-2 infection and provide new targets for the discovery of novel antiviral treatments against COVID-19.

## Results and discussion

### The cellular RNA-binding proteome globally responds to SARS-CoV-2 infection

**Figure 1:**
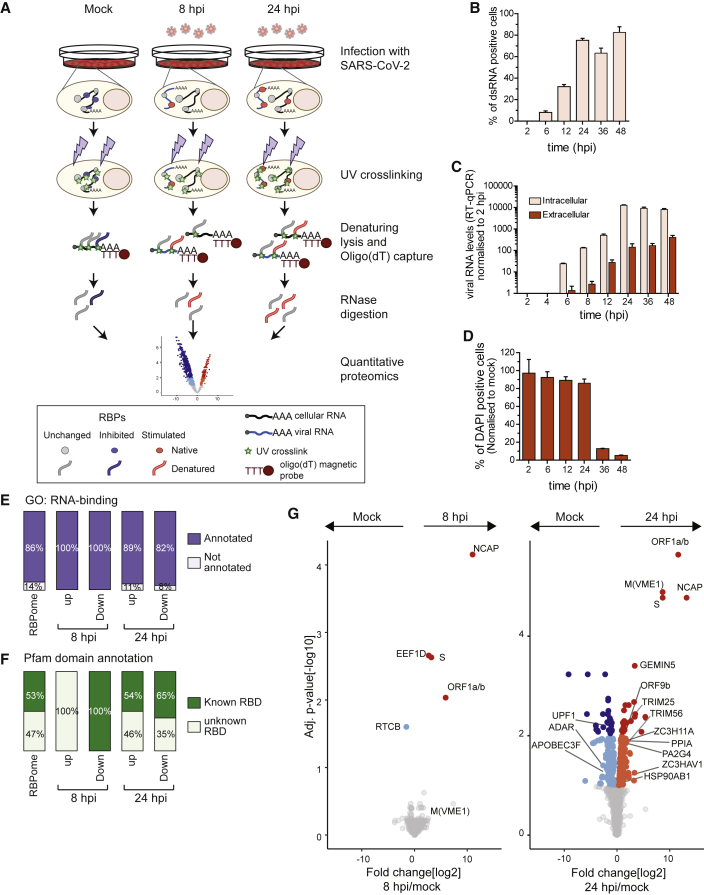
RBPome analysis in SARS-CoV-2-infected cells (A) Schematic representation of cRIC. (B) Proportion of infected cells estimated by immunofluorescence using an antibody against dsRNA. (C) qRT-PCR analysis of SARS-CoV-2 RNA in cells and in the supernatant of infected cells. (D) Number of adhered cells at different times after SARS-CoV-2 infection counted using a DAPI stain. (E) Proportion of RBPs that are annotated by the GO term RNA binding. (F) Proportion of RBPs that harbor known RBDs. (G) Volcano plots showing the log2 fold change (x axis) and its significance (adjusted [adj.] p value, y axis) of each protein (dots) in the cRIC experiments (n = 3). 1% FDR proteins are in blue and red; 10% FDR proteins in orange and cyan. Error bars in (B)–(D) represent the standard error of the mean (SEM); n = 3.

Cellular RBPs are fundamental for viruses, because they can promote or suppress infection. To elucidate the landscape of active RBPs in SARS-CoV-2-infected cells, we used cRIC (Garcia-Moreno et al., 2019). cRIC employs zero distance, UV protein-RNA crosslinking, followed by denaturing lysis, oligo(dT) selection of polyadenylated (poly(A)) RNA, and quantitative proteomics (Garcia-Moreno et al., 2019; Perez-Perri et al., 2021; Sysoev et al., 2016). To determine the optimal conditions for these experiments, we performed infection kinetics in epithelial human lung cancer cells (Calu-3). SARS-CoV-2 RNA and infective particles increase over time and peak at 24 h postinfection (hpi) (Figures 1B, 1C, and S1A). Subsequently, cell numbers sharply decrease from 36 hpi, suggesting widespread cell death (Figure 1D). We thus chose two stages of the viral life cycle: (1) an early time point at which viral RNA is exponentially increasing (8 hpi) and (2) a late time point at which viral RNA and extracellular virions peak (24 hpi), before cell death induction. cRIC was then applied to SARS-CoV-2-infected (8 and 24 hpi) and uninfected cells (Figure 1A). We identified 809 proteins, 86% of which are annotated by the Gene Ontology (GO) term RNA binding and are enriched in well-established RNA-binding domains, resembling previously established RBPomes (Figures 1E, 1F, and S1B; Table S1) (Hentze et al., 2018). 70 proteins displayed changes greater than 2-fold at 8 hpi, although only 5 qualified as statistically significant (Figure 1G; Table S1). This suggests that early RBP responses are either subtle or variable across replicates. Conversely, 335 RBPs were significantly altered at 24 hpi. Of these, 176 showed increased and 159 showed decreased RNA-binding activity (Figure 1G; Table S1). Importantly, SARS-CoV-2-regulation affects both classical RBPs and unorthodox RBPs lacking known RNA-binding domains (RBDs) (Figure 1F). Moreover, regulated RBPs, especially those stimulated by SARS-CoV-2, include proteins annotated by GO terms and KEGG pathways related to antiviral response and innate immunity (Figure S1C). Altogether, these results reveal that SARS-CoV-2 infection initially causes subtle remodeling of the cellular RBPome (8 hpi) that becomes pervasive by 24 hpi. cRIC also identified three viral RBPs at 8 hpi and five at 24 hpi (Figure 1G). These include known viral RBPs such as nucleocapsid (NCAP) and the polyprotein ORF1a/b, as well as proteins not known to interact with RNA, such as M, S, and ORF9b.

### Potential causes for SARS-CoV-2-induced RBPome remodeling

**Figure 2:**
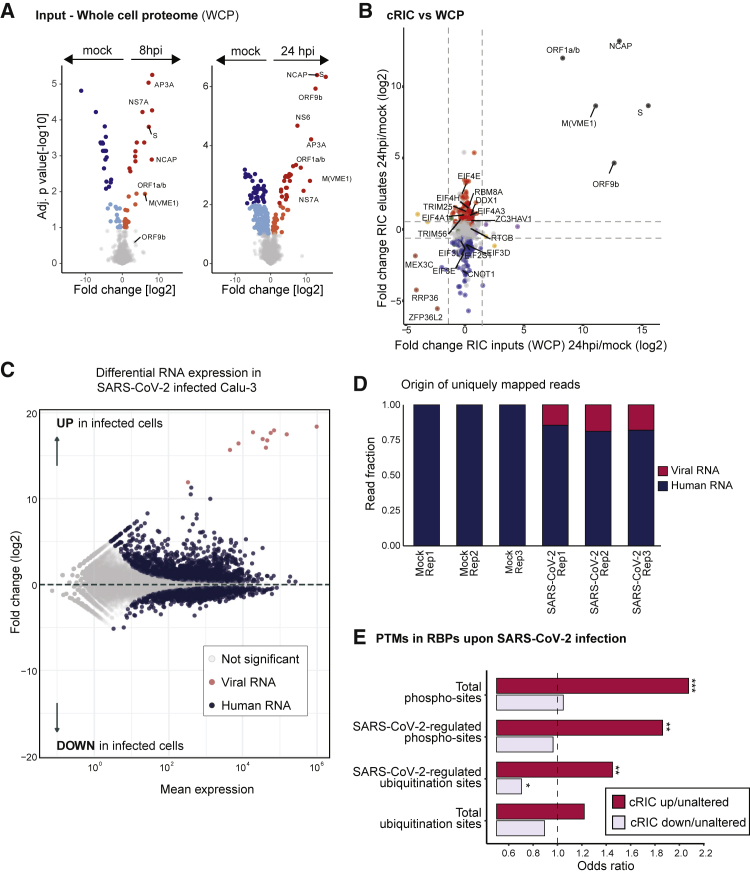
Factors influencing RBP remodeling in SARS-CoV-2-infected cells (A) Proteomic analysis of the whole-cell proteome (inputs) of the cRIC experiment. Volcano plots showing the log2 fold change and adjusted p value of each protein (n = 3). 1% FDR proteins are in blue and red; 10% FDR proteins in orange and cyan. (B) Scatterplot showing the fold changes in cRIC and those in the WCP. In red and blue are the RBPs upregulated or downregulated, respectively, in the cRIC experiment (FDR < 10%). (C) MA plot highlighting significant changes in gene expression in SARS-CoV-2-infected Calu-3 cells as detected by RNA sequencing (RNA-seq). (D) Fraction of uniquely aligned RNA-seq reads mapping to human chromosomes or the SARS-CoV-2 genome in uninfected and infected cells. (E) Bar plot showing the odds ratio of previously reported total and SARS-CoV-2 differentially regulated posttranslational modifications (PTMs) in upregulated and downregulated RBPs, relative to the non-regulated RBPs within the cRIC experiment. ^∗^p < 0.1; ^∗∗^p < 0.05; ^∗∗∗^p < 0.01.

We hypothesized that the remodeling of the RBPome induced by SARS-CoV-2 can simply be a consequence of changes in protein abundance, as previously reported for fruit fly embryo development (Sysoev et al., 2016). To assess this possibility, we analyzed the whole-cell proteome (WCP) of SARS-CoV-2-infected and uninfected cells (Figures 2A, 2B, and S2A–S2C; Table S2). 69 and 222 proteins of the 4,555 quantified proteins exhibited significant changes in abundance at 8 and 24 hpi, respectively (Figures 2A and S2D; Table S2). As expected, all viral proteins increased in abundance as infection progressed (Figure 2A). The WCP analysis covered 82% of the proteins identified by cRIC, providing an overview of RBP levels in infected and uninfected cells. When cRIC and WCP were compared, we observed correlation only for viral proteins and a few cellular proteins (Figure 2B). This reflects that the capture of viral proteins by cRIC increases as viral proteins accumulate. Conversely, changes in cRIC were not matched by similar changes in WCP for most RBPs (Figure 2B). These results stood when recently published WCP datasets were used (Klann et al., 2020; Stukalov et al., 2020), despite an increase of RBP coverage to 93% (Figures S2B and S2C). Lack of correlation between RBPome and WCP unequivocally indicates that protein abundance is not a global contributor to RBP responses in SARS-CoV-2-infected cells.

RNA abundance can also influence the RBPome, so we analyzed poly(A)-selected RNA sequencing data from Calu-3 cells infected with SARS-CoV-2 for 24 h (Blanco-Melo et al., 2020) (Figures 2C, 2D, and S2E–S2G). As expected, SARS-CoV-2 causes substantial alterations in the cellular transcriptome, with 5,465 RNAs displaying significant fold changes compared with the uninfected control (2,733 upregulated and 2,732 downregulated RNAs, with p < 0.01) (Figures 2C and S2E). Notably, viral RNAs emerge as the dominant poly(A) RNA species in the cell, representing 14%–19% of the reads (Figures 2C and 2D). These results have two major implications: (1) viral RNAs become new abundant substrates for cellular RBPs and (2) they are captured by oligo(dT) and thus must contribute to the changes observed by cRIC. Altogether, the alterations in cellular mRNA levels and the emergence of the viral RNA as the most abundant poly(A) RNA likely have a major impact on the composition of the RBPome in SARS-CoV-2-infected cells.

Posttranslational modifications (PTMs) are known to regulate RBPs (Arif et al., 2018; Castello et al., 2016). We hypothesized that SARS-CoV-2-induced PTMs can also affect RBP dynamics. To test this possibility, we used SARS-CoV-2-regulated PTMs from recently published datasets (Bouhaddou et al., 2020; Klann et al., 2020; Stukalov et al., 2020) and mapped these to cRIC-identified RBPs. Of the 335 RBPs regulated by SARS-CoV-2, 123 possessed differential phosphorylation sites and 62 possessed differential ubiquitination sites (Table S3). Strikingly, these SARS-CoV-2-regulated PTMs occur more frequently in upregulated RBPs than in downregulated or unaltered RBPs (Figure 2E), suggesting that PTMs could contribute to the RBP’s ability to interact with RNA. Indeed, we observed that SARS-CoV-2-modulated RBPs were more frequently phosphorylated at multiple sites than their unaltered counterparts (Figure S2H). These results suggest that posttranslational control may contribute to the differential RNA-binding activity observed for dozens of RBPs in SARS-CoV-2-infected cells. In summary, the combination of the changes in the transcriptome (Figures 2C and 2D) and posttranslational regulation (Figure 2E; Table S3) are likely contributing to the regulation of RBP activities reported here.

### Kinetics of RBP alterations upon SARS-CoV-2 infection

**Figure 3:**
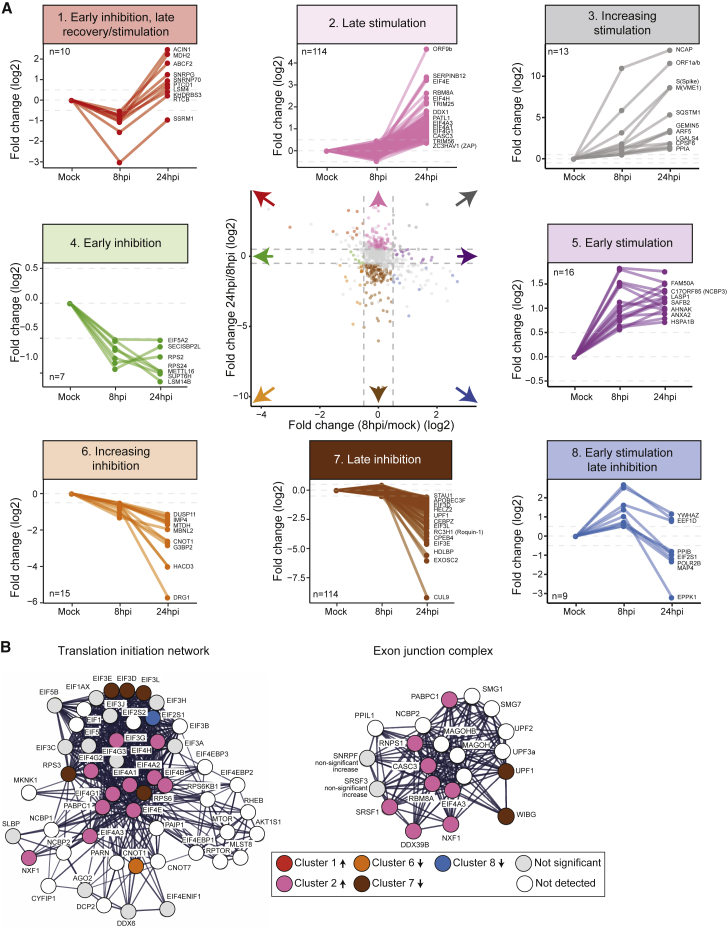
Clustering of RBP responses to SARS-CoV-2 infection (A) Cluster analysis of the RBP dynamics using data from uninfected cells, 8 hpi and 24 hpi. (B) Protein-protein interaction network of the translation initiation complex and the exon junction complex generated with STRING. Proteins are colored based on the cluster in (A).

The kinetics of RBP activation and inhibition can be informative for protein complex dynamics and function. To characterize RBP responses after SARS-CoV-2 infection, we clustered proteins based on their cRIC fold changes at 8 and 24 hpi. Our analysis distinguished eight RBP response profiles (Figure 3A; Table S4). Clusters 2 and 7 were dominant, with 114 proteins in each group, reflecting that most RBPs changes are only detected at 24 hpi. By contrast, 70 RBPs exhibited more complex RNA-binding patterns, distributing across clusters 1, 3, 4, 5, 6, and 8.

SARS-CoV-2 RNAs accumulate throughout the infection, and proteins involved in viral replication or its suppression may well display similar kinetics. Accordingly, cluster 3 is composed of RBPs whose RNA-binding activity increases throughout the infection. Apart from most viral RBPs, cluster 3 harbors several notable cellular factors that either have been linked to virus infection or are known to play critical roles in cellular pathways required for viruses. These include the antiviral protein GEMIN5 (Garcia-Moreno et al., 2019; Martinez-Salas et al., 2020), the autophagy factor SQSTM1 (p62) (Horos et al., 2019), and the master regulator of virus infection PPIA (cyclophilin A) (Dawar et al., 2017).

SQSTM1 (also p62) is a critical component of the autophagy pathway that plays a key role as a receptor of the autophagy substrates and mediates interaction with growing phagophores to form autophagosomes (Büscher et al., 2020). In a report, it was shown that SQSTM1 is inhibited by interaction with vault (vt) RNA1-1 (Horos et al., 2019). The interaction of SQSTM1 with RNA is mediated by its ZZ and PB1 domains, and the resulting complex is unable to mediate autophagy. The strong increase in RNA-binding activity of SQSTM1 upon SARS-CoV-2 infection suggests that autophagy is inhibited upon infection through this pathway. The vault complex, which contains vtRNAs, has been reported to reside close to the double-membrane vesicles that are the sites of viral replication (Klein et al., 2020). However, whether the increase in SQSTM1 RNA-binding activity involves vtRNA1-1 or viral RNA requires further investigation.

SARS-CoV-2 NSP1 inhibits protein synthesis by interacting with the ribosome’s mRNA channel (Banerjee et al., 2020; Schubert et al., 2020; Thoms et al., 2020). To determine how this inhibitory interaction affects cellular RBPs, we analyzed the kinetic profiles of all proteins annotated by translation and ribosome GO terms. We observed the presence of several components of eukaryotic initiation factor (EIF) 3, EIF2S1 (also EIF2α); elongation factors; and ribosomal proteins in clusters 4, 6, 7, and 8, which are composed of downregulated RBPs (Figures 3A and 3B, S3A, and S3B; Table S4). Conversely, the cap- and poly(A)-binding proteins eIF4E and PABPC1, as well as translation initiation factors such as EIF4A1, EIF4A2, EIF4B, EIF4G1, and EIF4G3, are present in cluster 2, which is composed of upregulated RBPs (Figures 3A and 3B; Table S4). These opposing results support a model in which the cap- and poly(A)-binding factors can interact with cellular mRNAs but cannot associate with EIF3 and the ribosomal subunit 40S, which agrees with the reported action of NSP1 preventing 40S recruitment to cellular mRNAs (Gehring et al., 2009; Schubert et al., 2020; Tidu et al., 2020; Yi et al., 2021).

If this model is correct, it is expected that the exon junction complex (EJC) would accumulate onto cellular mRNAs, because it is removed during the pioneering round of translation (Gehring et al., 2009; Yi et al., 2021). To test this hypothesis, we searched for the core components of the EJC in our dataset and observed that EIF4A3, RBM8A, and CASC3 are upregulated in SARS-CoV-2-infected cells (also in cluster 2) (Figures 3A, 3B, and S3E). Conversely, the EJC removal factor WIBG (PYM1) (Gehring et al., 2009) is downregulated, supporting that co-translational removal of EJCs is impaired in infected cells. Moreover, the crucial nonsense-mediated decay factor UPF1 (cluster 7) is inhibited upon infection, which reflects that co-translational quality control is not taking place efficiently. Collectively, these results indicate that SARS-CoV-2-induced protein synthesis shutoff may cause the accumulation of matured transcripts into a translation-inactive state.

Deposition of EJCs on cellular RNAs is a consequence of the splicing reaction (Yi et al., 2021). However, a recent study reported that NSP16 interacts with the U1 and U2 small nuclear RNAs (snRNAs) and disrupts splicing (Banerjee et al., 2020). To assess the effects of NSP16 in RBP dynamics, we examined the cRIC fold changes of all spliceosome-associated proteins. Surprisingly, the components of the core spliceosomal complexes showed no significant changes, except for SNRPG, which was substantially upregulated (Figure S3D; Table S1). Conversely, several splicing factors showed strong changes in RNA-binding activity, including the branchpoint binder U2AF2, U2SURP, most serine/arginine (SR)-rich splicing factors (SRSFs), and several HNRNPs (Figures S3D–S3F). Many of these proteins play important roles in exon and intron definition, as well as in the recruitment of the spliceosome (Ule and Blencowe, 2019). In agreement, we observed 786 differentially used exons in 560 genes at 24 hpi (Figure S2G). These results suggest that the alterations in splicing factors induced by SARS-CoV-2 infection may cause substantial effects in alternative splicing.

### Comparison of SARS-CoV-2- and SINV-induced alterations of the RBPome

**Figure 4:**
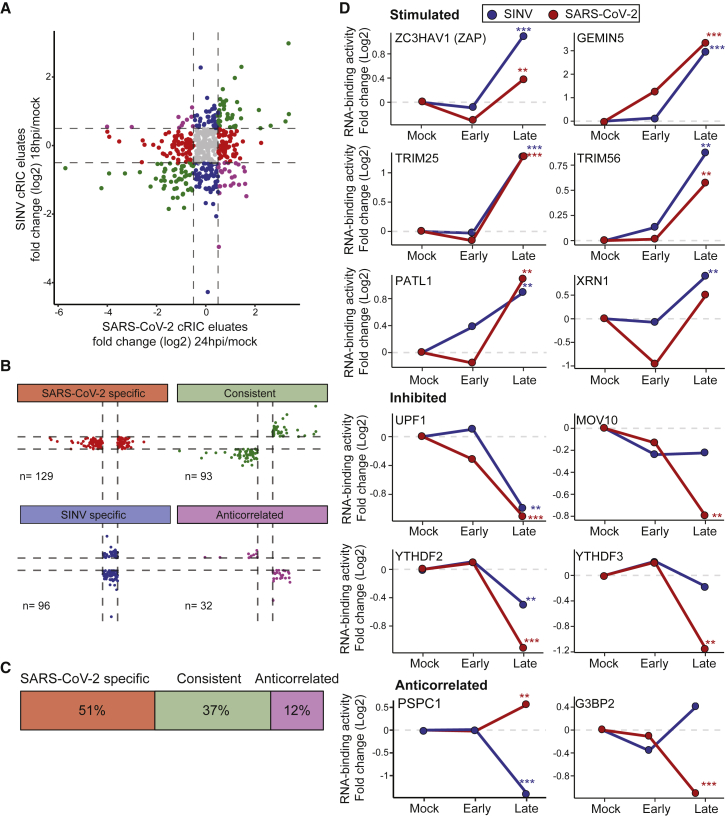
Analysis of RBP dynamics in SARS-CoV-2- and SINV-infected cells (A) Scatterplot of the fold change between infected and uninfected cells, using the data from the cRIC experiments in cells infected with SARS-CoV-2 (24 hpi) and SINV (18 hpi). (B and C) Proteins were grouped based on their behavior (B), and overlap of datasets was estimated (C). (D) Fold change of selected proteins in SARS-CoV-2 (red) and SINV (blue) cRIC analyses. Early and late are 8 and 24 hpi for SARS-CoV-2 and 4 and 24 hpi for SINV, respectively. ^∗^FDR < 20%, ^∗∗^FDR < 10%, ^∗∗∗^FDR < 1%.

To determine whether the changes that SARS-CoV-2 induces in the cellular RBPome are shared with other viruses, we compared the SARS-CoV-2 cRIC data with that of SINV (Garcia-Moreno et al., 2019). SINV is a positive stranded virus from the alphavirus genus. Like SARS-CoV-2, the SINV genome is capped and polyadenylated, although it is substantially smaller (∼11 versus ∼30 kb). Moreover, both viruses produce subgenomic RNAs and replicate in the cytoplasm. Strikingly, nearly 40% of the changes in RBP activity observed in SARS-CoV-2 were also present in the SINV cRIC dataset (Figures 4A–4C). This result indicates that even if these viruses belong to different families and have little or no sequence homology, they cause similar alterations in the RBPome that are consistent for both upregulated and downregulated RBPs (Figures 4A and 4B). Several antiviral factors were noticeable among the 93 RBPs, with consistent responses: TRIM25, TRIM56, ZC3HAV1 (also ZAP), DHX36, and GEMIN5 (Figures 4D and S4A). These antiviral RBPs are upregulated in both datasets, suggesting that they are likely involved in the antiviral response against both SARS-CoV-2 and SINV. TRIM25 is an E3 ubiquitin ligase whose catalytic activity is triggered by RNA binding and interacts with SINV RNA (Choudhury et al., 2017; Garcia-Moreno et al., 2019). TRIM25 antiviral activity is thought to be mediated by the ubiquitination of RIGI and ZC3HAV1/ZAP (Gack et al., 2007; Li et al., 2017). Although RIGI was not detected in our analysis, ZC3HAV1/ZAP RNA-binding activity was upregulated in response to infection, suggesting that it may be the effector activated by TRIM25. GEMIN5 is an antiviral factor that interacts with the cap and 5′ UTR of SINV RNA and suppresses viral mRNA translation (Garcia-Moreno et al., 2019; Martinez-Salas et al., 2020). Given that SARS-CoV-2 RNAs are also capped, it is thus plausible that GEMIN5 hampers SARS-CoV-2 gene expression following a similar mechanism. Other RBPs with prominent roles in virus infection were consistently upregulated by SARS-CoV-2 and SINV, including PPIA (cyclophilin A), PA2G4, ZC3H11A, DDX3, and HSP90AB1 (Figure S4B) (Dawar et al., 2017; Garcia-Moreno et al., 2019; Valiente-Echeverría et al., 2015; Younis et al., 2018).Our data also revealed antiviral RBPs that are downregulated by SARS-CoV-2 and, in several instances, by SINV. These include the RNA-editing enzymes ADAR, APOBEC3F, and APOBEC3G and the nonsense-mediated decay helicase UPF1 (Figure S4C).

Interestingly, 12% of the proteins exhibited opposite behavior in the two viral models. Many of these can be traced back to membraneless organelles such as paraspeckles and stress granules. The core paraspeckle components NONO, PSPC1, SFPQ, and MATR3 display opposite trends, being repressed by SINV and stimulated or unaffected by SARS-CoV-2 (Figures 4D and S4D). It is proposed that paraspeckles are critical to sequester proteins and/or mRNAs to regulate gene expression, although the importance of paraspeckle proteins in virus infection remains poorly understood (Fox et al., 2018). Similar anticorrelation was observed with the stress granule proteins G3BP1 and G3BP2 (Figures 4D and S4D). Stress granules play a defensive role against viruses by sequestering viral RNA (McCormick and Khaperskyy, 2017). Alphaviruses like SINV are known to suppress stress granule formation, and this is accompanied by an increase of G3BP1 and G3BP2 RNA-binding activity (Garcia-Moreno et al., 2019; Kim et al., 2016; Panas et al., 2012; Scholte et al., 2015). The inhibition of G3BP1 and G3BP2 in SARS-CoV-2-infected cells may thus reflect an opposite outcome, i.e., lower association with RNA because of the induction of stress granules.

### The SARS-CoV-2 RNA interactome

**Figure 5:**
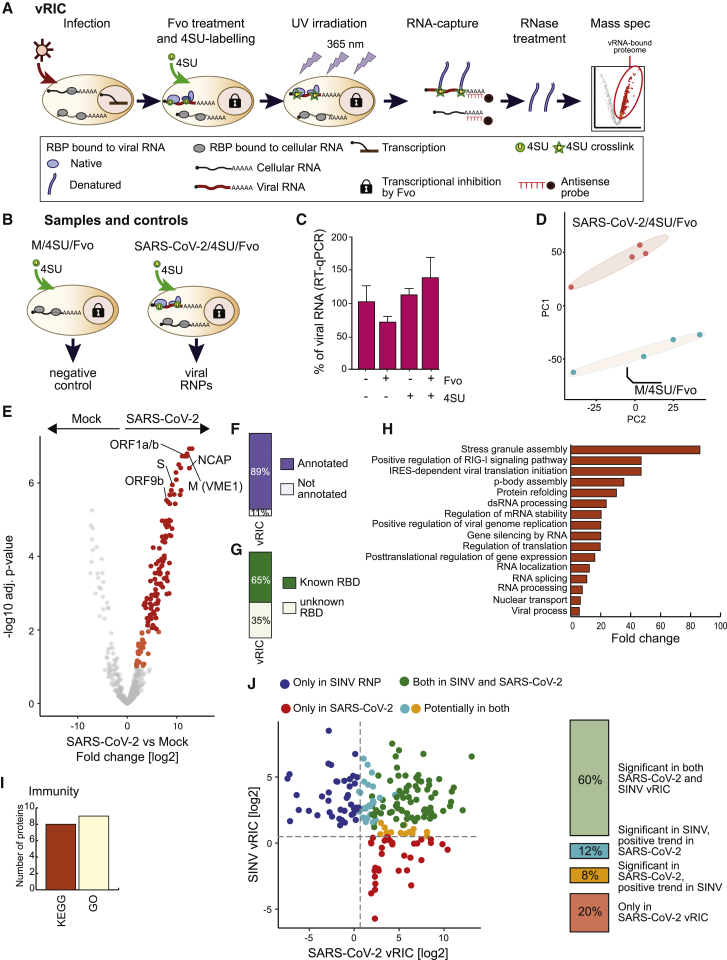
vRIC analysis of the SARS-CoV-2 RNA interactome (A) Schematic representation of vRIC. (B) Controls used in the vRIC experiment (expanded in Figure S5E). (C) Effects of 4SU and Fvo on SARS-CoV-2 RNA levels analyzed by qRT-PCR. Error bars represent SEM (n = 3). (D) Principal-component analysis (PCA) of vRIC in SARS-CoV-2-infected and uninfected cells (n = 4). (E) Volcano plots showing the log2 fold change and adj. p value of each protein in the vRIC experiment. 1% FDR proteins are in red, and 10% FDR proteins are in orange. (F and G) Proportion of the proteins enriched by vRIC that are annotated by the GO term RNA binding (F) or harbor classical RBDs (G). (H) GO enrichment analysis of the proteins enriched by vRIC. (I) Proportion of the proteins enriched by vRIC that are annotated to immunity in GO or KEGG. (J) Scatterplot showing the fold change between infected and uninfected cells, using the vRIC data from SARS-CoV-2- and SINV-infected cells. On the right, a boxplot shows the overlap of the two datasets.

cRIC captures both SARS-CoV-2 and cellular mRNAs, which represent 14%–19% and 80%–84% of the eluted RNA, respectively (Figures 2D and S2F). Therefore, it is not possible to know *a priori* which of the observed protein-RNA interactions are driven by viral RNA. To systematically identify the RBPs that interact directly with SARS-CoV-2 RNAs, we applied a newly developed approach that we named vRIC (Figures 5A, 5B, and S5A). SARS-CoV-2-infected and uninfected Calu-3 cells are treated with the RNA polymerase II (RNAPII)-specific inhibitor flavopiridol (Fvo), followed by a pulse with the photoactivatable nucleotide analog 4-thiouridine (4SU). Because viral RNA polymerases are insensitive to Fvo, temporal inhibition of RNAPII causes 4SU to be predominantly incorporated into nascent viral RNAs. Cells are then UV irradiated at 365 nm to induce crosslinks between viral RNA and proteins placed at a zero distance from the 4SU molecules. Because natural nucleotide bases do not absorb UV at 365 nm, protein-RNA crosslinking is restricted to 4SU-containing viral RNA. Cells are then lysed under denaturing conditions, and poly(A)-containing RNA is captured with oligo(dT), following a previously designed robust procedure (Castello et al., 2012). After elution, proteins co-purified with the viral RNA are analyzed by proteomics.

Our control experiments showed that Fvo strongly abrogates RNAPII transcription from a strong tetracycline-inducible cytomegalovirus promoter and that neither Fvo nor 4SU interfered with SARS-CoV-2 replication (Figures 5C and S5A–S5C). In mock cells, 4SU incorporation followed by 365 nm UV crosslinking and oligo(dT) capture led to the isolation of the steady-state RBPome (Figures S5E–S5I). However, when 4SU was omitted or Fvo was added, the amount of protein co-isolated with RNA was massively reduced in both silver staining and proteomic analyses (Figures S5D, S5F, and S5G). These results show that active RNAPII is required in uninfected cells to achieve efficient 4SU-dependent protein-RNA UV crosslinking. Conversely, when cells were infected with SARS-CoV-2, efficient protein isolation was observed despite Fvo treatment (Figures 5D, 5E, and S5E–S5I). These findings confirm that 4SU incorporation into nascent viral RNAs promotes effective UV protein-RNA crosslinking at 365 nm (Figures 5D, 5E, and S5E–S5I). In agreement, a principal-component analysis revealed that the datasets derived from uninfected and SARS-CoV-2-infected cells are clearly distinct (Figure 5D), with 139 RBPs enriched in vRIC eluates from SARS-CoV-2-infected cells (SARS-CoV-2/4SU/Fvo) over the mock control (M/4SU/Fvo); 107 had a 1% false discovery rate (FDR), and 32 additional proteins were at 10% FDR (Figure 5E; Table S5). The SARS-CoV-2 mRNA interactome is enriched in proteins annotated by the GO term RNA binding (89%) and harboring known RBDs (65%) (Figures 5F and 5G), supporting the capacity of vRIC to identify bona fide protein-RNA interactions. The SARS-CoV-2 RNA interactome is enriched in the following GO terms: associated RNA metabolism (RNA splicing, transport, stability, silencing, and translation), antiviral response (e.g., RIGI pathway), cytoplasmic granule assembly (stress granules and P bodies), and virus biology (e.g., viral process, dsRNA binding, and IRES-dependent viral RNA translation) (Figure 5H). Notably, 8 and 9 proteins were annotated by innate immunity-related terms in KEGG and GO, respectively (Figure 5I).

A complementary SARS-CoV-2 RNA interactome has been generated in SARS-CoV-2-infected hepatoma (Huh-7) cells using RAP-MS, which combines UV crosslinking and specific antisense probes (Schmidt et al., 2021). This dataset overlaps with our vRIC data, despite being generated with different cell types (hepatocytes versus lung epithelial cells) and methods (RAP-MS versus vRIC) (Figure S5J). However, vRIC identified substantially more RBPs than RAP-MS at all FDR cutoffs tested, providing additional SARS-CoV-2 RNA interactors.

To determine to what extent the SARS-CoV-2 RNA interactome harbors cellular RBPs that are also present in the RNPs of other viruses, we compared the SARS-CoV-2 vRIC to a SINV vRIC dataset generated in a parallel study (W.K., S.M., and A.C., unpublished data). The SARS-CoV-2 vRIC dataset is smaller than the SINV counterpart, likely because of the limited starting material available (Figure S5K). Nevertheless, 60% of RBPs within the SARS-CoV-2 RNA interactome were present in that of SINV (Figure 5J). These results suggest that viral RNPs may share a larger proportion of cellular factors than previously anticipated, opening the possibility to target commonly used RBPs in broad-spectrum therapeutic approaches.

The cRIC analysis revealed global alterations of the translation machinery (Figures 3B, S3A, and S3B). To test whether these alterations also apply SARS-CoV-2 RNAs, we examined the translation factors present in viral RNPs. Most proteins involved in the recognition of the cap and poly(A) tail are identified in SARS-CoV-2 RNP, including EIF4G1, EIF4G3, EIF4A1, EIF4A2, EIF4B, and PABPC1 (Figure 5E; Table S5). However, one of the critical components is missing: the cap-binding protein EIF4E. Although we cannot rule out that this missing protein is a false negative, other capped RNA viruses such as SINV can initiate translation without EIF4E, calling for further experiments to discriminate between these two possibilities (Carrasco et al., 2018). Moreover, several core EIF3 subunits (A, C, D, and G) are highly enriched in the SARS-CoV-2 RNP, revealing that the molecular bridge connecting the ribosome and the mRNA (Merrick and Pavitt, 2018) is active in SARS-CoV-2 mRNAs, despite the downregulation of several EIF3 subunits in the cRIC analysis (Figure 5E; Table S5). These results suggest that even though EIF3 subunits C and D have an overall reduced association with RNAs, likely due to NSP1 action, they interact with SARS-CoV-2 RNA to enable viral protein synthesis.

cRIC revealed upregulation of many HNRNPs (Figure S3F). To test whether viral RNA is involved in these alterations, we examined the vRIC dataset. Notably, 10 HNRNPs interact with SARS-CoV-2 RNA, particularly from the A family (A0, A1, A2B1, A3, C, DL, M, L, Q [SYNCRIP], and R). Immunofluorescence analysis revealed that a subpopulation of HNRNPA1 accumulates at cytoplasmic viral dsRNA-containing foci (Figure S5L). These results suggest that the enhancement of HRNP RNA-binding activity may be driven by SARS-CoV-2 RNA accumulation.

The cRIC analysis revealed a connection between SARS-CoV-2 infection and RNA granules (Figures 4D and S4D). To determine whether such interplay involves the viral RNA, we searched for known components of RNA granules in the vRIC dataset. We noticed the presence of core stress granule components G3BP1 and G3BP2 and their interacting proteins CAPRIN1, NUFIP2, and USP10 within SARS-CoV-2 RNPs (Figure 5E; Table S5). These results, together with the observed downregulation of G3BP1 and G3BP2 (Figures 4D, S4D, and S4E) and their interaction with the viral NCAP (Gordon et al., 2020), reflect an intimate relationship between stress granules and SARS-CoV-2 RNAs. In addition, the P-body components DDX6, LSM14A, and PATL1 and the miRNA mediator AGO2 interact with SARS-CoV-2 mRNA. Conversely, none of the nuclear paraspeckle proteins were statistically enriched in the viral RNP, suggesting that their role in SARS-CoV-2 infection, if any, might be indirect. Collectively, our data show that SARS-CoV-2 RNA engages with components of stress granules and P bodies.

SARS-CoV-2 RNA is posttranscriptionally edited, although the importance of this remains unknown (Kim et al., 2020b). To obtain more insights into this phenomenon and its consequences in the composition of the viral RNP, we searched for all editors and readers that interact with SARS-CoV-2 RNAs (Table S5). ADAR is downregulated upon SINV infection (Table S1); however, it is highly enriched in SARS-CoV-2 RNPs (Figure 5E; Table S5). It catalyzes the conversion of adenosines to inosine, which can affect several aspects of RNA function, including structure, RBP-binding sites, and coding sequence, potentially regulating viral replication. The participation of ADAR in SARS-CoV-2 infection is underscored by a recent study reporting adenosine deamination in the SARS-CoV-2 RNA (Di Giorgio et al., 2020). Methyl 6 adenosine (m6A) also plays critical roles in virus infection, and viral RNA is typically enriched with this modification (Tan and Gao, 2018). m6A is recognized by a family of proteins known as readers, which regulate RNA fate (Wang et al., 2014). Although the readers YTHDF2 and YTHDF3 are downregulated in both SINV- and SARS-CoV-2-infected cells, YTHDC1 and YTHDC2 are stimulated (Figures 4D, S4F, and S4G). These opposed results indicate that m6A readers are differentially regulated in response to infection. Our vRIC analysis shows that YTHDC2 is significantly enriched in the SARS-CoV-2 RNPs (Figure 5E; Table S5). These results support the potential role of YTHDC2 and perhaps YTHDC1 as mediators of m6A function in SARS-CoV-2 infection.

Other proteins that interact with SARS-CoV-2 RNA include five helicases (DDX1, DDX3X, DDX6, DDX60, and DHX57); five chaperones (HSP90AA1, HSP90AB1, HSPA5, HSPA8, and HSPB1); the actin-interacting proteins SYNE1 and SYNE2; the vesicle membrane protein VAT1, which interacts with M, ORF7b (NS7B), and ORF9b (Gordon et al., 2020); the antiviral protein OASL, which belongs to a family of SARS-CoV-2 susceptibility factors (Pairo-Castineira et al., 2020); and three separate subunits of the protein phosphatase 1 (PPP1CA, PPP1CB, and PPP1R3A). Collectively, vRIC shows that SARS-CoV-2 RNA engages with a range of cellular RBPs, including classical and unconventional RNA binders.

### Viral proteins that interact with viral and cellular RNA

**Figure 6:**
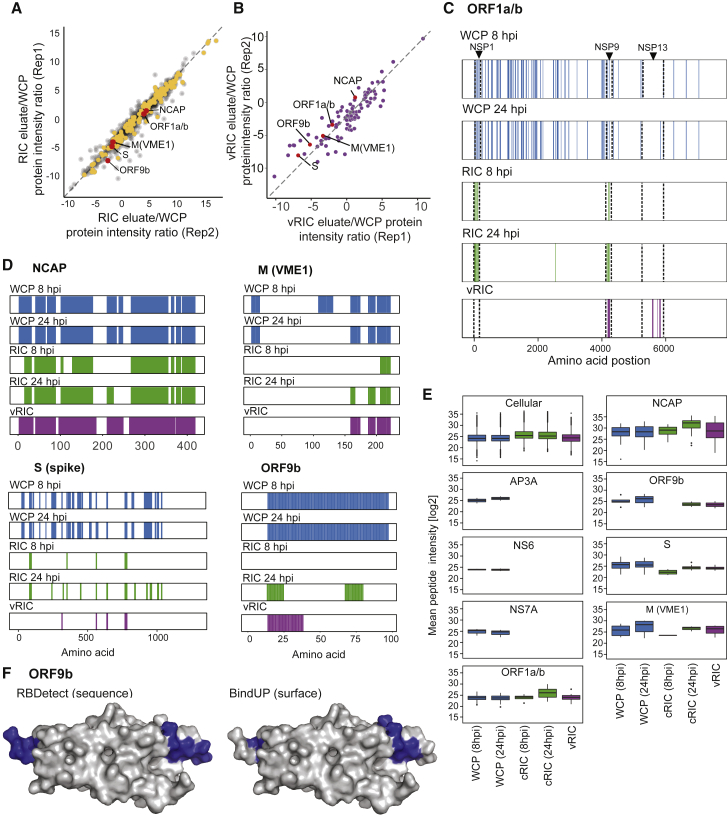
SARS-CoV-2 proteins that interact with RNA (A and B) Representative scatterplot showing the cRIC (left, 24 hpi/mock) or vRIC (right, infected/uninfected) fold change normalized to the fold change in the WCP (24 hpi/mock). Replicates 1 and 2 were chosen as illustrative examples; remaining comparisons can be found in Figure S6A. Cellular RBPs upregulated in the cRIC experiments (Figure 1G) are in yellow, cellular RBPs enriched in SARS-CoV-2 vRIC (Figure 5E) are in violet, and viral proteins are in red. (C and D) Sequence coverage analysis. Peptides detected in WCP (blue), cRIC (green), and vRIC (violet) are mapped to the viral proteins plotted from N terminus to C terminus (x axis). (E) Boxplot showing peptide intensity distribution in cRIC, vRIC, and WCP for each of the viral proteins detected. Colors as in (C) and (D). (F) ORF9b structure showing the protein surface (PDB: 6z4u). Peptides with a high probability of RNA binding by RBDetect (left) or BindUP (right) are in blue.

Both cRIC and vRIC agree in the behavior of SARS-CoV-2 proteins that interact with RNA, even though these methods employ different crosslinking chemistries (Castello et al., 2012). Viral RBPs include the polyprotein ORF1a/b, NCAP, and surprisingly, M, S, and ORF9b (Figures 1E, 1F, 5E, 6A, 6B, and S6A). To determine which type of interaction these proteins establish with RNA, we normalized the protein intensity in vRIC and cRIC by that of the WCP (Figures 6A, 6B, and S6A; Table S6). NCAP and ORF1a/b displayed the highest UV “crosslink-ability,” followed by M, ORF9b, and S. Generally, the efficiency of crosslinking depends on several factors, including (1) the geometry of the protein-RNA interaction (contacts with the nucleotide bases), (2) the physicochemical properties of the bases and amino acids in close proximity, (3) the duration of the interaction, and (4) the proportion of the protein that engages in RNA binding. We can thus suggest that ORF1a/b and NCAP establish optimal and stable interactions with RNA, whereas M, ORF9b, and especially S mediate shorter-lived and/or geometrically less favorable interactions for crosslinking. However, the high protein sequence coverage and peptide intensity in both vRIC and cRIC experiments strongly support that all these proteins interact with viral RNA (Figures 6C–6E and S6B).

ORF1a/b is a polyprotein comprising of 16 mature polypeptides. Although the peptides detected in the WCP mapped uniformly throughout the polyprotein, both vRIC- and vRIC-identified peptides clustered only in specific regions (Figure 6C). The first peptide cluster mapped to NSP1 and was only detected by cRIC (both 8 and 24 hpi). The lack of signal in vRIC samples strongly indicates that NSP1 interacts notwith viral RNA but with cellular mRNAs, which are highly enriched by oligo(dT) (Figure S2F). Similarly, the SARS-CoV-2 RNA interactome from Schmidt et al. (2021) detected NSP1 with a single peptide with close-to-noise intensity levels, and (Tidu et al., 2020) did not detect interaction with viral RNA by *in vitro* electrophoretic mobility assays. Thus, although NSP1 appears to promote selective translation of viral RNAs, this regulatory effect seems not to involve a direct interaction with them. The second peptide cluster mapped to NSP9 and is present in both vRIC and cRIC (Figure 6C). The detection of NSP9 by vRIC agrees well with its known role in viral replication and the well-established interaction of its SARS-CoV-1 ortholog with single-stranded RNA (Chandel et al., 2020; Egloff et al., 2004). The third peptide cluster mapped to the RNA helicase NSP13, which is critical for SARS-CoV-2 replication (Chen et al., 2020). Cluster 3 peptides are only detected by vRIC, which supports that NSP13 only interacts with viral RNA.

The proteins M and S also reliably and robustly co-purify with RNA upon cRIC and vRIC (Figures 1E, 1F, 5E, 6A, 6B, 6E, and S6B). The most likely scenario in which these proteins could engage with viral RNA is during virus assembly and within viral particles (Klein et al., 2020; Yao et al., 2020). To determine whether M and S have sequences compatible with RNA binding, we used RBDetect, a software package that predicts RBDs based on amino acid sequence. Strikingly, we detected two segments in the intravirion region of M that share sequence similarities with bona fide RNA-binding sites present in cellular RBPs (Figure S6C). Similarly, the intravirion part of S also harbors a ∼15 amino acid motif compatible with RNA binding (Figure S6C). Both M and S RNA-binding regions are present in both SARS-CoV-2 and SARS-CoV-1, suggesting that the underlying functions are conserved. Although we cannot fully rule out that these interactions with RNA are stochastic because of protein-RNA proximity in the context of the virion, their prominence in the vRIC and cRIC data suggest that they may play a role in infection (Figures 6C–6E and S6B). For example, they may contribute to the recruitment of viral RNA or to the budding and/or structural arrangement of the viral particle. NCAP clusters locate underneath the viral envelope during budding of the viral particles, and this structure persists in the mature particles (Klein et al., 2020; Yao et al., 2020). Cryo-electron tomography analysis of infected cells revealed that membrane invagination at the budding site appears to require the presence of NCAP (Klein et al., 2020), implying a potential role for RNA in the process of particle formation.

The viral protein ORF9b was also consistently identified by both cRIC and vRIC, supporting that it is a novel RNA-binding protein (Figures 1E, 1F, 5E, 6A, and 6B). Little is known about ORF9b beyond its ability to interfere with interferon responses (Jiang et al., 2020). To determine whether ORF9b also contains sequences compatible with RNA binding, we used RBDetect (sequence-based software). Given the availability of a deposited structure (6Z4U) (Weeks et al., 2020), we also considered surface physicochemical properties (BindUP) (Paz et al., 2016). Both approaches agree that there is a discrete region in ORF9b that generates a positively charged surface with high probability to interact with nucleic acids (Figures 6G, S6C, and S6D). Further work is required to define the role of the RNA-binding activity of ORF9b in SARS-CoV-2 infection.

Therefore, our data reveal seven viral proteins that harbor RNA-binding activity, six of which interact with SARS-CoV-2 RNA. Among these, M, S, and ORF9b emerge as novel RBPs based on both our study and Schmidt et al. (2021).

### Functional importance of cellular RBPs in SARS-CoV-2 infection

**Figure 7:**
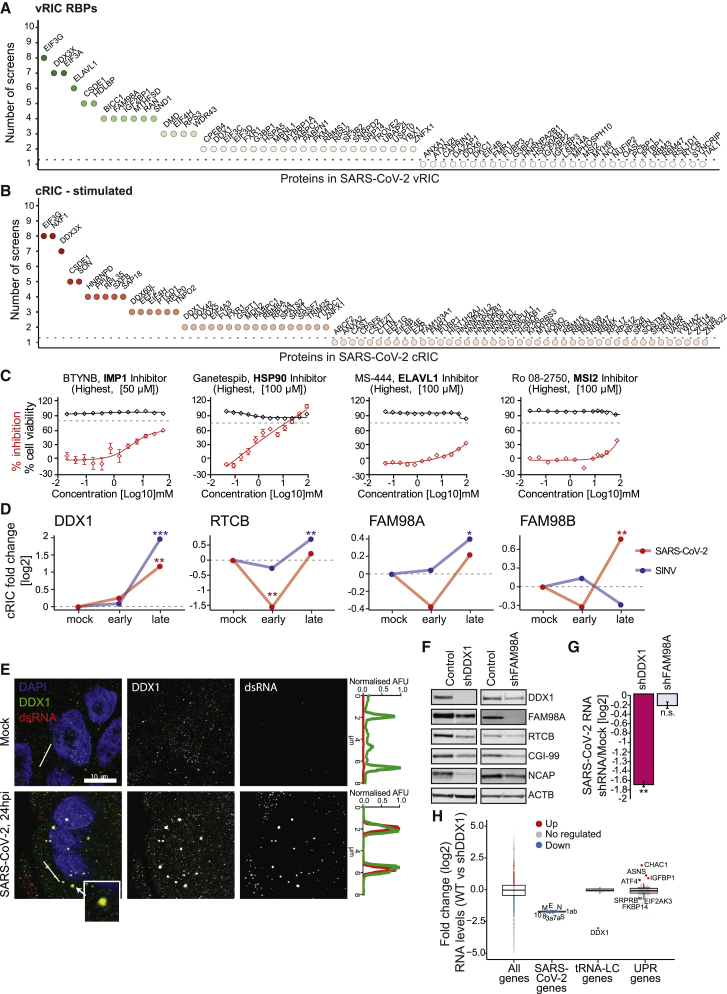
Functional characterization of protein-RNA interactions in SARS-CoV-2-infected cells (A and B) Proteins with identified phenotypes in genome-wide screens using viruses. RBPs enriched in SARS-CoV-2 vRIC (A) or upregulated in the cRIC experiment (B) are displayed along the x axis. The y axis indicates the number of screens in which the protein has caused a phenotype in infection. (C) Effects of RBP inhibitors on SARS-CoV-2 infection. The red line indicates the effects in infection measured by protein ELISA at each drug dose. The black line shows cell viability at each drug dose. Error bars are SEM from three independent experiments. (D) RNA-binding profiles of the components of the tRNA ligase complex in cells infected with SARS-CoV-2 (red) and SINV (blue) (as in Figure 4D). ^∗^FDR < 20%, ^∗∗^FDR < 10%, and ^∗∗∗^FDR < 1%. (E) Confocal immunofluorescence images of SARS-CoV-2 and mock-infected Calu-3 cells using antibodies against DDX1 and dsRNA. Fluorescence plot shows green and red fluorescence intensity profiles across an 8 μm section (white line). (F) Western blot analysis showing the nucleocapsid (NCAP), components of the tRNA-LC complex, and β-actin (ACTB) levels in control cells and upon DDX1 or FAM98A knockdown. (G) SARS-CoV-2 RNA levels in control cells and upon DDX1 or FAM98A knockdown measured by qRT-PCR and normalized to β-actin mRNA. Error bars are SEM from three independent experiments. (H) RNA-seq analysis of wild-type (WT) and DDX1 knockdown A549-ACE2 cells infected with SARS-CoV-2. Data are represented in a boxplot, showing different mRNA groups (all, SARS-CoV-2, tRNA-LC, and UPR genes). Significant changes (p < 0.05) are shown in red and blue.

To determine whether our study has potential for the discovery of new regulators of SARS-CoV-2 infection, we assessed the incidence of vRIC- and cRIC-identified proteins in genome-wide screens with other viruses. The superset includes studies using RNA interference (RNAi), CRISPR-Cas9, and haploid line screens for 36 viruses (Table S7). This analysis revealed that cRIC and vRIC identified 47 RBPs linked to phenotypes in functional screenings (>3 studies) (Figures 7A, 7B, and S7A; Table S7). Moreover, we used an automated PubMed search pipeline to assess how many RBPs have been robustly linked to virus infection in the literature. 73 (43.5%) RBP upregulated in cRIC, 51 (32.5%) RBP downregulated in cRIC, and 67 (51.1%) RBPs detected by vRIC were already linked to virus infection (Figure S7B). These results indicate that our dataset is rich in regulators of viral infection.

To determine the biomedical potential of cellular RBPs for COVID-19 treatment, we compared the subset of RBPs stimulated by SARS-CoV-2 infection and the subset of proteins that interact with SARS-CoV-2 RNA to drug databases (Figure S7C). Importantly, 54 proteins within these datasets have potential inhibitors available (Figure S7C). To prove the value of these RBPs as therapeutic targets, we tested five drugs in Calu-3 cells infected with SARS-CoV-2 (Figures 7C and S7D). Our results show that two of these compounds targeting HSP90 and IGF2BP1 (IMP1) cause a strong inhibition of SARS-CoV-2 protein production, with two additional drugs targeting ELAVL1 (HuR) and MSI2 causing moderate effects and one compound targeting PKM having slight effects. The anti-SARS-CoV-2 effects of HSP90 inhibitors have been recently confirmed by an independent study (Wyler et al., 2021). These results reflect the potential of RBPs as targets for antiviral drugs.

### The tRNA ligase complex, a new regulator of SARS-CoV-2 infection

vRIC revealed DDX1, RTCB, and FAM98A as components of the SARS-CoV-2 viral ribonucleoprotein (vRNP). These proteins, together with FAM98B, C14ORF166 (CGI99 and CLE), and C2ORF49 (ASW), form the tRNA ligase complex (tRNA-LC) (Popow et al., 2011). DDX1, RTCB, FAM98A, and FAM98B interact directly with RNA and are regulated by both SARS-CoV-2 and SINV infection. Although DDX1 displayed a continuous increase in RNA-binding activity in the cRIC experiment, the other proteins follow an early-inhibition and late-increase pattern (Figure 7D). The tRNA-LC mediates the ligation of unusual RNA fragments, one with 3′-phosphate or 2′,3′-cyclic phosphate and the other a 5′-hydroxyl group (Popow et al., 2011, 2014). Only a few endonucleases can cleave RNA in this way, including the endoplasmic reticulum resident protein IRE1, which is activated in response to unfolded protein response (UPR) (Jurkin et al., 2014; Popow et al., 2011). Viruses are known to cause UPR, suggesting that they should activate the endonuclease IRE1 (Galluzzi et al., 2017). UPR leads to the tRNA-LC-dependent cytoplasmic splicing of *Xbp1* mRNA, which encodes a critical transcription factor that coordinates the cellular responses to UPR (Jurkin et al., 2014).

The regulation of the RNA-binding components of the tRNA-LC by SARS-CoV-2 infection and their presence in viral RNPs suggest their involvement in the viral life cycle. To confirm the interaction between tRNA-LC and SARS-CoV-2 RNA, we performed an immunofluorescence analysis of infected Calu-3 cells. DDX1, a core component of the tRNA-LC, concentrates at the cytoplasmic foci where dsRNA accumulates, confirming that DDX1 engages with SARS-CoV-2 RNA.

To test the relevance of the tRNA-LC in SARS-CoV-2 infection, we generated A549-ACE2 cells with tetracycline-inducible expression of short hairpin RNA (shRNAs) against DDX1 and FAM98A. Knocking down DDX1 led to the depletion of other components of the tRNA-LC, including the ligase RTCB, FAM98A, and to a lesser extent, CIG-99 (Figures 7F and S7E). These results support previous observations showing that the stability of the tRNA-LC relies on presence of the core subunits of the complex (Jurkin et al., 2014). Knocking down the peripheral member of the tRNA-LC, FAM98A, causes minor effects in the levels of the other components (Figure S7E). Silencing DDX1 caused a strong reduction of intracellular SARS-CoV-2 RNA that correlates with a parallel reduction of NCAP (Figures 7E, 7F, and S7E). FAM98A knockdown (KD) led to milder effects in both viral RNA levels and NCAP accumulation (Figures 7F, 7G, and S7E). Because DDX1 is a core subunit of the tRNA-LC and FAM98A is secondary, these differential effects are expected. To provide further insights into the effects of DDX1 KD, we generated RNA-seq data. We observed that DDX1 KD equally affected all viral transcripts, despite having no effect on cell viability (Figure 7H). Conversely, DDX1 KD had no detectable effects in *xbp1* mRNA expression and splicing, and with few exceptions, most UPR response genes remained unaltered (Figures 7H and S7G–S7J). This indicates either that DDX1 KD leaves sufficient tRNA-LC for the *xbp1* mRNA splicing to occur or that SARS-CoV-2 does not produce a strong UPR response in A549-ACE2 cells. These results suggest that tRNA-LC plays a role in SARS-CoV-2 replication.

### Outlook

We provide a systematic and comprehensive analysis of protein-RNA interactions in SARS-CoV-2-infected cells. We show that SARS-CoV-2 infection induces a pervasive remodeling of the RBPome, which involves the upregulation and downregulation of more than 300 RBPs. We also discovered dozens of cellular proteins that interact with SARS-CoV-2 RNAs, which are promising for the development of new therapeutic approaches. We find shared host-virus interactions between SARS-CoV-2 and SINV that reflect the existence of cellular RBPs with master regulatory roles in virus infection. Similar work with other viruses and cell types will expand our knowledge on these critical protein-RNA interactions. The relevance and complementarity of our datasets are illustrated by the discovery of the tRNA-LC as a key regulator of SARS-CoV-2, as well as RBP-targeting compounds with antiviral activity. Our study also discovers novel viral RBPs, including S, M, and ORF9b, opening new angles to investigate their roles in SARS-CoV-2 infection.

In the future, cRIC and vRIC could be extended to other coronaviruses and other biological models, such as primary cells and organoids. Generating additional time points and using replication inhibitors such as remdesivir, it will be possible to study the dynamics of viral RNPs throughout the infection. Moreover, combining such approaches with CLIP-based methods will make it possible to identify the motifs that cellular RBPs recognize in viral RNAs and will provide new insights into their function in infection. We are hopeful that this work will shed light on the pathogenesis of SARS-CoV-2 and accelerate the discovery of therapies for COVID-19.

### Limitations of the study

Like any proteomic approach, RIC and vRIC have a bias related to protein abundance, size, and physicochemical properties of their tryptic peptide sequences. UV irradiation induces RNA-to-protein crosslinks in a specific manner, because it requires zero distances. However, the higher specificity comes at the price of lower efficiency compared with chemical crosslinkers such as formaldehyde. UV underperforms with transitory interactions and contacts with the ribose-phosphate backbone as the crosslinking is mediated by the nucleotide base (Castello et al., 2016). These biases may explain why a few proteins within ORF1a/b that have been linked to viral RNA metabolism are not identified by vRIC. In the case of vRIC, the combination of 4SU and Fvo could potentially have undesired effects, and we recommend titrating both compounds to avoid/minimize side effects in cell viability and virus fitness (Figures S5A–S5D). Here, we combined vRIC with oligo(dT) capture; however, it is compatible with virtually any RNA isolation approach, including specific antisense probes or total RNA isolation approaches. These alternatives must be evaluated when working with non-polyadenylated viruses.

## STAR★Methods

### Key resources table

**Table:** 

REAGENT or RESOURCE	SOURCE	IDENTIFIER
**Antibodies**		

Fam98A	Aviva	ARP55 265_P050; RRID:AB_2045839
Tubulin	Sigma	T9026; RRID:AB_477593
CGI-99(C14orf166)	Atlas antibodies	HPA039824; RRID:AB_10793922
dsRNA	Jena Bioscience	RNT-SCI-10010200; RRID:AB_2651015
SARS-CoV2 Nucleocapsid	Sino Biological	AB 40143-MM05; RRID:AB_2827977
HNRNPA1	Cusabio	CSB-PA00109A0Rb
rabbit Alexa Fluor 488	Thermo Fischer	#A-11008; RRID:AB_143165
mouse Alexa Fluor 647	Thermo Fischer	#A-21235; RRID:AB_2535804
DDX1 (immunofluorescence)	Cambridge Bioscience Ltd	HPA034502; RRID:AB_10794321
DDX1 (immunoblotting)	Bethyl	A300-521A; RRID:AB_451046

**Bacterial and virus strains**		

SARS-CoV-2 hCoV-19/England/02/2020	Public Health England propagated viral isolate Feb 2020	EPI_ISL_407073
SARS-CoV-2 hCoV-19/Germany/BavPat1/2020	European Virology Archives: 026V-03883	EPI_ISL_406862

**Chemicals, peptides, and recombinant proteins**		

Ganetespib	BIOZOL	BYT-ORB181166
MS-444	Hycultec	HY100685-1mg
compound 3k	BIOZOL	SEL-S8616
Ro 08-2750	TOCRIS	2272
BTYNB	Cayman Chemical	25623

**Deposited data**		

Proteomic data	This study	PRIDE: PXD023418
NGS data	This study	GEO: GSE171382
NGS data	Blanco-Melo et al., 2020	GEO: GSM4462348 to GEO: GSM4462353

**Experimental models: Cell lines**		

Calu-3	kind gift from Dr. Manfred Frey, Mannheim, Germany	N/A
A549-Ace2	Klein et al., 2020	N/A

**Software and algorithms**		

DESeq2 (1.28.1)	Love et al. (2014)	N/A
ggrepel 0.8.2	(Slowikowski, 2020) https://github.com/slowkow/ggrepel	N/A
GO.db 3.11.4	Carlson, 2019	N/A
htseq*-*count 0.11.3	(Anders et al., 2015) https://github.com/simon-anders/htseq/releases	N/A
KEGGREST 1.28.0	Tenenbaum and Maintainer, 2021	N/A
limma 3.38.3	Ritchie et al. (2015)	N/A
org.Hs.eg.db 3.11.4	Carlson, 2019	N/A
PerformanceAnalytics 2.0.4	(Peterson and Carl, 2020) https://github.com/braverock/PerformanceAnalytics	N/A
PFAM.db 3.11.4	Carlson et al., 2019	N/A
RBDetect	https://nishuai.shinyapps.io/RBDetect/	N/A
rentrez 1.2.2	(Winter, 2017) https://github.com/ropensci/rentrez/releases	N/A
scales 1.1.1	(Wickham and Seidel, 2020) https://scales.r-lib.org/	N/A
SRA toolkit	SRA Toolkit Development Team. http://ncbi.github.io/sra-tools/	N/A
Stats 4.0.2	R core team https://www.R-project.org/	N/A
tidyverse suite 1.3.0	(Wickham et al., 2019) https://tidyverse.org	N/A
viridis 0.5.1	(Garnier, 2018) https://github.com/sjmgarnier/viridis	N/A
VSN 3.50.0	Huber et al. (2002)	N/A
DEP 1.4.1	Zhang et al. (2018)	N/A

### Resource availability

#### Lead contact

Further information and requests for resources and reagents should be directed to and will be fulfilled by the Lead Contact, Alfredo Castello (alfredo.castello@glasgow.ac.uk).

#### Materials availability

Material is available upon request from the authors.

#### Data and code availability

The mass spectrometry proteomics data have been deposited to the ProteomeXchange Consortium via the PRIDE partner repository with the dataset identifier PRIDE: PXD023418. The accession number for the RNA sequencing data reported in this study is GEO: GSE171382.

### Experimental model and subject details

#### Cell culture

Calu-3 cells (kind gift from Dr. Manfred Frey, Mannheim, Germany) were maintained in DMEM (GIBCO, 41965039) with 20% fetal bovine serum (FBS) (GIBCO, 10500064) and 1x penicillin/streptomycin (Sigma Aldrich, P4458) at 37**°**C with 5% CO_2_. A549-Ace2 (Klein et al., 2020) were maintained as above with 10% FBS. Both cell lines are male. To generate inducible knockdown lines, cells were infected with Lentiviral vectors derived from pLKO-Tet-On (Wiederschain et al., 2009) with the guide sequence GATGTGGTCTGAAGCTATTAA for DDX1 and GCACATTCAGTAGCCTTATTT for FAM98A. Lentiviruses were produced by co-transfection of HEK293T cells with pHEF-VSVG (NIH AIDS Research & Reference reagent program #4693) and psPAX2 (kind gift N. Proudfoot, Oxford, UK). After infection of A549-Ace2 cells with the lentiviruses, selection was performed with 1 μg/ml. shRNAs were induced by addition of 1ug/ml doxycycline.

#### Viruses

Infection of Calu-3 cells for virus growth kinetics, cRIC, vRIC, WCP and drug screen was performed using isolate hCoV-19/Germany/BavPat1/2020 (European Virology Archives: 026V-03883, EPI_ISL_406862). For validation in knockdown studies and immunofluorescence, hCoV-19/England/02/2020 (Public Health England propagated viral isolate Feb 2020, EPI_ISL_407073) was used.

### Method details

#### Virus growth kinetic experiments

1.2 × 10^5^ Calu-3 cells were seeded into each well of a 24-well plate. Cells were infected 24 hours after seeding with SARS-CoV-2 at a multiplicity of infection (MOI) of 1. To determine infectivity, 50 μl of supernatant from each well was used in plaque assays. Plaque assays were performed as previously described (Klein et al., 2020). Briefly, 2.5 × 10^5^ Vero cells were seeded into each well of a 24-well plate and cells were inoculated with 10-fold serial dilutions of SARS-CoV-2 containing supernatants for 1 h at 37°C. After 1h, viral supernatants were replaced by serum-free MEM (GIBCO #11095080, Life Technologies) containing 0.8% carboxymethylcellulose (Sigma, 11095080). Three days later, plates were fixed with 6 % formaldehyde for 30 minutes and rinsed with tap water. Plates were stained with a solution containing 1% crystal violet (Sigma, HT90132-1L) and 10% ethanol for 30 min. After rinsing with tap water, plaques were counted to determine viral titer.

For intra- and extra-cellular RNA extraction, NucleoSpin RNA extraction kit (Macherey-Nagel, #740955.50) was used following the manufacturer’s specifications. cDNA synthesis from the total RNA isolated was achieved using a high-capacity reverse transcription kit (ThermoFisher, #4368814). cDNA samples were diluted 1:15 and used for qPCR with the iTaq Universal SYBR green mastermix (Biorad, #1725120). Cycle threshold values were corrected for PCR efficiency of each primer set and normalized to the hypoxanthine phosphoribosyltransferase 1 (HPRT) mRNA to determine relative abundance of viral RNA for each sample (see Table S8).

#### Cell viability assay and determination of infection rate

To establish cell viability and infection rate, 1.2 × 10^5^ Calu-3 cells were seeded into each well of a 24-well plate onto glass coverslips. Mock-infected and SARS-CoV-2-infected cells were fixed at the times post infection indicated in the figures with 6% formaldehyde for 30 min. Cells were washed twice with PBS (phosphate-buffered saline) and permeabilized with 0.2% Triton X-100 in PBS. Permeabilized samples were incubated with blocking solution (2% of milk and 0.02% Tween-20 in PBS) for 1 h at room temperature. Samples were stained with primary antibodies specific to dsRNA (see Table S8) as well as DAPI (DAPI Fluoromount-G, SouthernBiotech, 0100-20) to visualize the nuclei using a Nikon Eclipse Ti microscope (Nikon, Tokio, Japan). Three replicates per time point were analyzed. Nuclei were counted with a custom-made macro for the Fiji software package (Schindelin et al., 2012). Number of nuclei in infected samples were normalized to the non-infected control counterparts. To determine the infection rate, the number of infected cells at each time point was determined using the dsRNA fluorescence signal with Fiji software using a custom macro (Schindelin et al., 2012).

#### Cell viability assay of knockdown cell lines

A549-Ace2 cells and the derived shRNA cell lines were cultured in doxycycline containing media (1 μg/ml) to induce shRNA expression for > 14 days. 5x10^4^ cells per condition were transferred into a 96-well plate. 24 hours later, ATP levels were measured using CellTiter-Glo 2.0 (Promega #G9241) on a BMG CLARIOStar Plus.

#### Colorimetric cell-based assay to assess the effects of RBP inhibitors in SARS-CoV-2 infection

Calu-3 cells were seeded at 2 × 10^4^ cells per well of 96-well plate. Cells were treated 24 hours later with 2-fold serial dilutions of the indicated compounds in duplicate wells. Dilutions ranged from 2.5 nM to 50 μM for Ro 08-2750 (TOCRIS, #2272) and the BTYNB IMP1 inhibitor (Cayman Chemical, #25623), 5 nM to 100 μM for Ganetespib (BIOZOL, BYT-ORB181166) and MS-444 (Hycultec, HY100685-1mg) and 1,25 nM to 25 μM for the PKM2 inhibitor - compound 3k (BIOZOL, SEL-S8616)). 2 hours after treatment, cells were infected with SARS-CoV-2 (BavPat1/2020 strain) at a MOI of 2. At 24 h post infection, plates were fixed with 6% formaldehyde for 30 min. Cells were then washed twice with PBS (Phosphate-buffered Saline) and permeabilized with 0.2% Triton X-100 in PBS. Permeabilized samples were then incubated with blocking solution (2% of milk and 0.02% Tween-20 in PBS) for 1 h at room temperature. Blocking solution was replaced with primary antibodies specific for SARS-CoV NCAP (Table S8) diluted in blocking solution. Cells were incubated for 1 h at 37°C, washed four times with PBS followed by incubation with horse radish peroxidase (HRP)-conjugated secondary antibodies diluted in PBS (containing 0.02% Tween-20) for 1 h at 37°C. Wells were washed 3 times with PBS. PBS excess was carefully removed, and wells were developed by adding 50 μl of TMB Microwell Peroxidase (SeraCare, Cat: 5120-0077) to each well for 5 min followed by 50 μl of 0.5 M H_2_SO_4_ solution to stop the reaction. Absorbance was measured at 450 nm using a Tecan-Sunrise absorbance microplate reader. Values were normalized to vehicle (DMSO). In order to assess the effects of the above-mentioned inhibitors on cell viability, we employed the commercial kit CellTiterGlo® Luminescent Cell Viability Assay (Promega, Cat: G7570) on a Mithras LB 940 plate reader (Berthold Technologies). The assays were performed following the manufacturer’s instructions in uninfected cells for the different doses of each compounds. Luminiscence values were normalized to vehicle (DMSO).

#### Immunofluorescence

Round #1.5 (diameter 13 mm) coverslips (Thermo Fischer Scientific) were wiped with lint-free tissue soaked in 80% ethanol and washed in 100% ethanol twice for 2 h. 2x10^5^ Calu-3 cells were seeded on the dried coverslips and incubated in growth media for 48 hours prior to the experiment. Cells were infected with 2x10^5^ PFU/well (MOI = 1) SARS-CoV-2 (hCoV-19/England/02/2020) and incubated for 24 hours. Cells were fixed in 4% formaldehyde for 30 minutes and washed once with PBS. Cells were permeabilised for 10 min with PBSTx (1x PBS + 0.1% Triton X-100) at room temperature. Next, cells were washed twice in PBSTw (1x PBS + 0.1% Tween-20) for 5 min each and incubated in blocking solution (PBSTw + 2.5% goat serum + 2.5% donkey serum) for 1 h at room temperature. Cells were incubated overnight at 4°C with primary antibodies diluted in blocking solution (Table S8). Coverslips were then washed three times with PBSTw for 10 min each at room temperature and incubated with secondary antibodies and DAPI (1 μg/ml) diluted in blocking solution overnight at 4°C. Cells were washed three times with PBSTw for 10 min each, once in PBS for 10 min, once in milliQ H_2_O and the coverslips were mounted on glass slides using Vectashield HardSet mounting medium (Vector Laboratories #H-1400). Mounted cells were imaged on an Olympus SoRa spinning disc confocal with Orca Flash4 CMOS camera using 100x silicone oil objective (1.35 NA, UPLSAPO100XS). Specimens were imaged in at least six different locations per coverslip. 3D-stacked images were taken with voxel size of 80 nm x 80 nm x 200 nm in x:y:z and images were deconvolved with maximum likelihood algorithm using cellSens (5 iterations, default PSF, no noise reduction, Olympus). Background subtraction was performed on all channels using rolling ball subtraction method (radius = 250 px) in ImageJ (National Institutes of Health). Fluorescence intensity profiles were obtained using ImageJ “Plot profile” tool across 8 μm regions on 0.4 μm max intensity z-projected images. Voxel intensities were normalized to maximum intensity value obtained from ‘SARS-CoV-2 infected’ condition.

#### qRT-PCR and RNA sequencing of knockdown lines

To induce shRNA expression A549-Ace2 cells and the derived shRNA lines were cultured in doxycycline containing media (1 μg/ml) for > 14 days. 2.5x10^5^ cells each were seeded into a 24-well plate and Cells were infected with 2x10^4^ PFU/well (MOI = 0.1) of SARS-CoV-2 (hCoV-19/England/02/2020). At 24 hpi, cells were detached and lysed in Trizol LS. Total RNA extraction was performed following manufacturers recommendation. qRT-PCR was performed using Luna (NEB # E3005L) with gene specific primers (Table S8). RNA sequencing libraries were prepared using the Illumina Total RNA Prep with Ribo-Zero Plus library kit (Cat# 20040525) according to manufacturer’s guidelines. Briefly, 100ng of total RNA was first depleted of the abundant ribosomal RNA present in the samples by rRNA targeted DNA probe capture followed by enzymatic digestion. Samples were then purified by Beckman Coulter RNAClean XP beads (Cat #A63987). Obtained rRNA-depleted RNA was fragmented, reverse transcribed, converted to dsDNA, end repaired and A-tailed. The A-tailed DNA fragments were ligated to anchors allowing for PCR amplification with Illumina dual indexing primers (Cat#20040553). Libraries were pooled in equimolar concentrations and sequenced on an Illumina NextSeq 500 sequencer using a high-output cartridge (Cat# 20024907), generating single 150bp long reads.

#### Comparative RNA interactome capture

Comparative RNA interactome capture (cRIC) was performed based on the previously described protocol (Castello et al., 2013; Perez-Perri et al., 2021) with the following alterations: Calu-3 cells were grown in sets of 3x15 cm dishes with 10^7^ cells/dish. One set of dishes remained uninfected while a second set was infected with SARS-CoV2 (hCoV-19/Germany/BavPat1/2020) at a MOI of 1. One of these infected cell sets was incubated for 8 h and the other for 24 h. 3 biological replicates for each condition were performed. After incubation, plates without lids were placed on ice and cells were irradiated with 150 mJ/cm^2^ of UV light at 254 nm and lysed with 5 mL of lysis buffer (20 mM Tris-HCl pH 7.5, 500 mM LiCl, 0.5% LiDS wt/vol, 1 mM EDTA, 0.1% IGEPAL (NP-40) and 5 mM DTT). Lysates were homogenized by passing the lysate at high speed through a 5 mL syringe with a 27G needle, repeating this process until the lysate was fully homogeneous. Ten percent of the lysate was separated for total proteome analysis (WCP). The rest of the samples were processed as follows. Protein content was measured using Qubit protein assay (Invitrogen Q33212) and lysates were normalized by protein content. 0.45 mL of pre-equilibrated oligo(dT)25 magnetic beads (New England Biolabs, #S1419S) were added to the lysates and incubated for 1 h at 4°C with gentle rotation. Beads were collected in the magnet and the lysate was transferred to a new tube and stored at 4°C. Beads were washed once with 5 mL of lysis buffer, followed by two washes with 5 mL of buffer 1 (20 mM Tris-HCl pH 7.5, 500 mM LiCl, 0.1% LiDS wt/vol, 1 mM EDTA, 0.1% IGEPAL and 5 mM DTT), and two washes with buffer 2 (20 mM Tris-HCl pH 7.5, 500 mM LiCl, 1 mM EDTA, 0.01% IGEPAL and 5 mM DTT), in all cases incubating the beads for 5 min at 4°C with gentle rotation. Beads were then washed twice with 5 mL of buffer 3 (20 mM Tris-HCl pH 7.5, 200 mM LiCl, 1 mM EDTA and 5 mM DTT) at room temperature for 3 minutes. Beads were resuspended in 300 μL of elution buffer and incubated for 3 min at 55°C with agitation. After collecting the beads with a magnet, eluates (supernatants) were collected and stored at −80°C. The lysates were subjected to a second round of capture and the eluates from the first and second cycles were combined. Prior to mass spectrometry sample processing, samples were RNase treated with ∼0.02U RNase A and RNase T1 at 37°C for 1h.

#### Viral RNA interactome capture

Viral RNA interactome capture was performed as in W.K., S.M., and A.C., unpublished data. Briefly, Calu-3 cells were grown in sets of 2x15 cm dishes. For the infected samples (SARS-CoV2/4SU/Fvo), at 8hpi (hours post-infection, MOI = 1), the growth media were replaced with fresh media supplemented with (20 μM Flavopiridol hydrochloride hydrate (Fvo, Cat.No. F3055, Sigma-Aldrich)) and 100 μM 4-Thiouridine (4SU, Cat.No. T4509, Sigma-Aldrich)). The plates were returned to the incubator for additional 16 hours. At 24hpi, growth media were discarded, and the cells were rinsed once with PBS (Phosphate-buffered saline). Cells were irradiated twice with at 200 mJ/cm^2^ using ultraviolet light 365nm. At this stage, samples were subjected to the standard RNA-interactome capture described above. For the control uninfected samples (M/4SU/Fvo), cells were treated as in SARS-CoV-2/4SU/Fvo with exception of not adding the virus. Both M/4SU/Fvo and SARS-CoV-2/4SU/Fvo were performed in sets of four biological replicates. Additional controls, (M/4SU/-), uninfected cells were treated as in M/4SU/Fvo, without the addition of Fvo, and (M/−/−) uninfected cells were incubated with growth media (not supplemented with Fvo and 4SU) and not crosslinked. Both (M/4SU/-) and (M/−/−) were performed in sets of three biological replicates.

#### Mass spectrometry

Prior to MS sample preparation, WCP samples were treated with benzonase for 30 min at room temperature to degrade both RNA and DNA. The cRIC, vRIC and WCP protein samples were processed via the bead-based single-pot, solid-phase-enhanced sample-preparation (SP3) method, using Speed Bead Magnetic Carboxylate Modified Particles (Sigma-Aldrich, cat.no.45152105050250) (Hughes et al., 2019). Protein digestion was performed using Trypsin Gold (MS grade; Promega, cat. no. V5280). Processed peptides were acidified by formic acid (final concentration 5%) prior to Mass spectrometry analysis.

For cRIC and vRIC peptides, liquid chromatography (LC) was preformed using Ultimate 3000 ultra-HPLC system (Thermo Fisher Scientific). Peptides were initially trapped in C18 PepMap100 pre-column (300 μm inner diameter x 5 mm, 100A, Thermo Fisher Scientific) in Solvent A (Formic acid 0.1% (v/v), Medronic acid 5 μM). Trapped Peptides were separated on the analytical column (75 μm inner diameter x 50cm packed with ReproSil-Pur 120 C18-AQ, 1.9 mm, 120 A, Dr. Maisch GmbH) in a 60min 15%–35% [vol/vol] acetonitrile gradient with constant 200 nL/min flow rate. Eluted peptides were directly electrosprayed into a QExactive mass spectrometer (Thermo Fisher Scientific). Mass spectra were acquired in the Orbitrap (scan range 350-1500 m/z, resolution 70000, AGC target 3 × 10^6^, maximum injection time 50 ms) in a data-dependent mode. the top 10 most abundant peaks were fragmented using CID (resolution 17500, AGC target 5 x10^4^, maximum injection time 120 ms) with first fixed mass at 180 m/z.

Both WCP and vRIC peptides were analyzed using a Dionex Ultimate 3000 RSLC nanoUPLC (Thermo Fisher Scientific Inc, Waltham, MA, USA) system online with an Orbitrap Eclipse mass spectrometer (Thermo Fisher Scientific Inc, Waltham, MA, USA). Peptides were loaded onto a trap-column (Thermo Scientific PepMap 100 C18, 5 μm particle size, 100A pore size, 300 μm i.d. x 5mm length) and separation of peptides was performed by C18 reverse-phase chromatography at a flow rate of 300 nL/min and a reverse-phase nano Easy-Spray column (Thermo Scientific PepMap C18, 2 μm particle size, 100A pore size, 75 μm i.d. x 50cm). WCP peptides were acquired in a 120 min run while vRIC samples in an 82 min run. Analytical chromatography for WCP peptides consisted of Buffer A (0.1% formic acid in HPLC-grade water) and Buffer B (80% ACN, 0.1% formic acid). 0-3 min at 2% buffer B, 3-90 min linear gradient 2% to 40% buffer B, 90-90.3 min linear gradient 40% to 90% buffer B, 90.3-95 min at 90% buffer B, 95-95.3 min linear gradient 90% to 2% buffer B and 95.3-120 min at 2% buffer B. Analytical chromatography for vRIC peptides was Buffer A (HPLC H_2_O, 0.1% formic acid) and Buffer B (80% ACN, 0.1% formic acid). 0-3 min at 3.8% buffer B, 3-63 min non-linear gradient 3.8% to 40% buffer B, 63-63.3 min linear gradient 40% to 90% buffer B, 63.3-68 min at 90% buffer B, 68-68.3 min non-linear gradient 90% to 3.8% buffer B and 68.3-82 min at 3.8% buffer B. All *m/z* values of eluting peptide ions were measured in an Orbitrap mass analyzer, set at a resolution of 120 000 and were scanned between *m/z* 380-1500 Da. Data dependent MS/MS scans (3 s duty cycle time) were employed to automatically isolate and fragment precursor ions using Collisional-induced Dissociation (CID) (Normalized Collision Energy of 35%). Only precursors with charge between 2 to 7 were selected for fragmentation, with an AGC target and maximum accumulation time of 1 × 10^4^ and 125 ms respectively. Precursor isolation was performed by the quadrupole with 1.2 m/z transmission window. MS2 fragments were measured with the Ion Trap analyzer. Dynamic exclusion window was set to 70 s.

Protein identification and quantification were performed using Andromeda search engine implemented in MaxQuant (1.6.3.4) under default parameters (Cox et al., 2011). Peptides were searched against reference Uniport datasets: human proteome (Uniprot_id: UP000005640, downloaded Nov2016) and SARS-CoV-2 (Uniprot_id: UP000464024, downloaded 24June2020). False discovery rate (FDR) was set at 1% for both peptide and protein identification. For cRIC and WCP samples, MaxQuant search was performed with “match between run” activated. For vRIC samples, since each sample was analyzed on both Eclipse and QExactive mass spectrometers, raw spectra form both runs were combined as separate fractions in the MaxQuant search (the spectra from the Eclipse was assigned fraction 1 and the spectra from the QExactive is assigned fraction 5, and each sample was as independent experiment).

### Quantification and statistical analysis

#### Proteomic quantitative analysis

For relative quantification, MaxQuant outputs (proteinGroups) were used for downstream analysis. Proteins flagged as potential contaminants were filtered out, using R-package “DEP (1.4.1)” (Zhang et al., 2018), together with proteins with all missing values. In case of cRIC and WCP experiments, proteins raw intensities were normalized and transformed using R-package Variance Stabilizing Normalization “VSN (3.50.0)” (Huber et al., 2002). Correlation analysis between replicates was preformed using R-package “PerformanceAnalytics (v2.0.4).” Missing value imputation was only preformed for proteins with missing values in all replicates in one experimental condition, while present in the other condition (at least in 2 out of 3 replicates). Imputation was preformed using local (by sample) minimum determination method (Mindet) (Lazar et al., 2016). Statistical analysis for the processed intensities was performed in R-package “limma (3.38.3)” (Ritchie et al., 2015) using empirical Bayesian method moderated t test. P values were adjusted for multiple-testing using Benjamini-Hochberg method. For the vRIC experiments, samples were processed as described above with exception of the normalization step.

#### Clustering of cRIC responses to SARS-CoV-2 infection

Cellular RBP responses to SARS-CoV-2 infection was classified into initial response, which is defined as cRIC log fold change from mock to early time point post-infection (8 hpi/mock), and progressive response, which is determined by log fold change from early to late time point post-infection (8 hpi/24 hpi). Protein abundance fold changes in these two stages were visualized using a scatterplot. The RNA-binding activities of cellular RBPs were divided into 8 clusters based on their initial response, progressive response, and FDR. Clustering was based on an FDR < 10% with a log(2) fold change of 0.5 as thresholds. For clustering of spliceosome/ spliceosome–related proteins, list of different classes of spliceosomal proteins was obtained from Spliceosomedb (Cvitkovic and Jurica, 2013).

#### SARS-CoV-2 proteins RNA binding prediction

RNA binding prediction for regions on the viral protein sequence was performed with RBDetect. RBDetect is a machine learning model trained by Shrinkage Discriminant Analysis (SDA) with a dataset of 8891 experimentally identified polypeptides from the RBDmap experiment, using positive examples (RNA-bound polypeptides) and negative examples (RNA released polypeptides) (Castello et al., 2016). For each amino acid position on the viral protein sequence, RBDetect assigns a probability value to bind RNA based on the fragment centered at that position. Then, a Hidden Markov model is used to visualize the probabilities in a sequential manner, which helps to determine the most probable binding regions on a larger scale.

#### Analysis of the PTM profile of RBPs identified in cRIC

Cellular RBPs detected in cRIC experiments were cross-referenced to phosphorylation and ubiquitination sites of recent large scale (post-translation modification) PTM quantification experiments preformed in SARS-CoV-2 infected cells. PTM datasets obtained from the single-time point phospho-proteome work by Klann et al. (2020), multi-time points phospho-proteome work by Bouhaddou et al. (2020), and multi-level omics work by Stukalov et al. (2020). The SARS-CoV-2 regulated RBPome is defined as RBPs with FDR < 0.1 in the cRIC experiment. SARS-CoV-2 regulated PTM sites are significant hits in each data sources using the criteria defined in the corresponding publications. For the multi-time point dataset, a PTM site is considered SARS-CoV-2 regulated, if it is determined as significant at any time point. Fisher’s exact test was employed to calculate odds ratios and significance of enrichment of each PTM annotation in the SARS-CoV-2 upregulated RBPome versus downregulated RBPome.

#### Drug-protein interactions

Cellular RBPs (stimulated upon SARS-CoV-2 infection (from cRIC) or bound to viral RNA (from vRIC)) were examined for known chemical compound interactions through the Drug-Gene Interaction database (DGIdb, downloaded Oct-2020) (Cotto et al., 2018).

#### Gene Ontology (GO) terms

Using the GO annotation available via the GO.db R package (3.11.4), GO terms including the term ‘RNA binding’ (to annotate RNA-binding related functions, processes, or compartments) or term ‘immun’ or exact terms ‘immune response’ and ‘innate immune response’ (to annotate immunity related functions, processes, or compartments) were selected. The full list of terms is provided as a supplementary table (Table S9). The R package org.Hs.eg.db (3.11.4) was used to identify the genes (proteins) in our dataset that are annotated to these GO terms using the cross-database id mapping functionality. GO enrichment analysis was performed using PANTHER classification system (http://www.pantherdb.org) (Mi et al., 2019).

#### Kyoto Encyclopedia of Genes and Genomes (KEGG) pathways

KEGG pathways under the ‘Immune system’ category in the high-level KEGG hierarchy available via the R package “KEGGREST” (1.28.0) were selected (see Table S9) and genes mapping to these pathways were identified using “org.Hs.eg.db.”

#### Pfam RNA-binding domains

Classification of proteins into classical and non-classical RNA-binding proteins is based on their Pfam domain composition. We considered RRM, KH, DSRM, Piwi, DEAD, PUF, CSD, and zf-CCCH domains as classical. These were obtained from the PFAM.db R package (3.11.4). Furthermore, we considered as non-classical RNA-binding domains those Pfam-A domains robustly identified as RNA-binding by RBDmap with at least 3 peptides and RNA interactome capture (Castello et al., 2012, 2016). The classification is provided in Table S9. The proteins containing these domains were identified using org.Hs.eg.db.

#### PubMed literature linking genes to viral infections

To automatically query the NCBI Entrez Utilities REST API, the R package “rentrez” (1.2.2) was used. For each gene symbol in our dataset the number of PubMed articles matching with a search query “(SYMBOL) AND (virus)” where SYMBOL is the gene name, such as EIF4E were retrieved. A minimum of five search results was considered a substantiated indication of a gene having a connection to virus-related literature.

#### RNA sequencing analysis

Strand-specific, poly(A) RNA-seq corresponding to SARS2-infected (MOI = 2) Calu-3 cells and controls from published work (Blanco-Melo et al., 2020) were downloaded from the Sequence Read Archive using “SRA toolkit “(2.10.8). Specifically, we analysed the following samples: Calu3 Mock 1 (GEO GSM4462348, SRA series SRX8089276, SRA run SRR11517744), Calu3 Mock 2 (GEO GSM4462349, SRA series SRX8089277, SRA run SRR11517745), Calu3 Mock 3 (GEO GSM4462350, SRA series SRX8089278, SRA run SRR11517746), Calu3 SARS-CoV-2 1 (GEO GSM4462351, SRA series SRX8089279, SRA run SRR11517747), Calu3 SARS-CoV-2 2 (GEO GSM4462352, SRA series SRX8089280, SRA run SRR11517748), Calu3 SARS-CoV-2 3 (GEO GSM4462353, SRA series SRX8089281, SRA run SRR11517749). Additionally, we processed the in-house generated RNA-seq in SARS2-infected WT and shDDX1 cells as detailed above. Raw reads alignment was performed via “STAR aligner” (2.7.3a) (Dobin et al., 2013), with splicing-aware settings, against human reference genome (GRCh38.99) and SARS-CoV-2 (NC_045512.2). Only uniquely aligned reads were used for downstream analyses. Mapped reads (exonic regions) counting was performed by “htseq*-*count” (0.11.3) in a strand-specific fashion. In order to assess the main driver(s) of variations across the RNA-seq samples, we performed a principal component analysis (PCA). First, we performed library size correction and variance stabilization with regularized–logarithm transformation implemented in “DESeq2” (1.28.1) (Love et al., 2014). This corrects for the fact that in RNA-seq data, variance grows with the mean and therefore, without suitable correction, only the most highly expressed genes drive the clustering. The 500 genes showing the highest variance were used to perform PCA using the “prcomp” function implemented in the base R package “*stats”* (4.0.2). Finally, differential expression analysis was performed using the R package “DESeq2” (1.28.1). “DESeq2” estimates variance-mean dependence in count data from high-throughput sequencing data and tests for differential expression based on a model using the negative binomial distribution.

#### Differential exon usage analysis

We performed differential exon usage analysis using the DEXSeq R package (1.34.1) (Anders et al., 2012) to assess changes in transcript isoforms between SARS2-infected and uninfected cells (three replicates each). Briefly, we created a flattened exon annotation from protein coding transcripts of genes and lncRNAs using the *dexseq_prepare_annotation.py* python script accompanying the package. We then assigned the reads into this simplified annotation using *dexseq_count.py* provided with DEXSeq in a strand-specific fashion. Data was then tested for differential exon usage and estimated exon fold changes using the R package. DEXSeq models count data using a negative binomial (NB) distribution and generalized linear models (Anders et al., 2012). We considered exons that did not overlap multiple genes with adjusted p value < 0.05 and absolute log2 fold change of at least 1 as significant.

#### tRNA-ligase component and unfolded protein response genes

We used the GO.db (3.11.4) and org.Hs.eg.db (3.11.4) Bioconductor R packages to identify GO terms and genes involved in tRNA splicing (GO:0072669, GO:0006388) and unfolded protein response (GO terms containing words ‘response to unfolded protein’ or ‘unfolded protein response’, 21 terms in total).

#### Xbp1 splicing and coverage

To plot Xbp1 coverage and splice junction usage, we used RSamtools (2.4.0), IRanges (2.22.2), GenomicRanges (1.40.0) and GenomicAlignments (1.24.0) Bioconductor R packages to read and identify reads mapping to region on chromosome 22 corresponding to *xbp1* gene, to calculate coverage in this region, and identify split reads spanning exon-exon junctions. To assess splicing at the *xbp1* exon determining the main protein isoform (positions 28,796,122 and 28,796,147 on chromosome 22), we identified reads that were spliced at these sites (resulting in long protein isoform) and those that were not (shorter protein isoform).

#### Data wrangling and visualization

The “tidyverse suite” (1.3.0) was used for data wrangling in R, and “rtracklayer” (1.48.0) for manipulating gtf annotation files (Lawrence et al., 2009). Furthermore, we used the following R packages in creating the presented visualization: “ggplot2*”* (3.3.2), “viridis*”* (0.5.1), “ggrepel*”* (0.8.2), “scales” (1.1.1).

## Supplemental information

Document S1. Figures S1–S7

Table S1. Analysis of RBP dynamics in SARS-CoV-2-infected cells by cRIC, related to Figure 1 cRIC datasets were generated at 8 and 24 hpi.

Table S2. Analysis of the total proteome of SARS-CoV-2-infected or mock-infected cells, related to Figure 2 WCP analyses were generated at 8 and 24 hpi.

Table S3. Posttranslational modification mapping to cellular RBPs, related to Figure 2 The cRIC data was cross-referenced to phospho-proteomic and ubiquinomic datasets reported by Bouhaddou et al. (2020), Klann et al. (2020), and Stukalov et al. (2020).

Table S4. Classification of RBPs by RNA-binding responses to SARS-CoV-2 infection, related to Figure 4 The different protein clusters were obtained by comparing the 8hpi/mock and 24hpi/8hpi fold-change of each RBP.

Table S5. vRIC analysis of the SARS-CoV-2 RNA interactome, related to Figure 5

Table S6. cRIC and vRIC normalization to the WCP, related to Figure 6 This table provides a ‘cross-link-ability’ index that can be used to classify RBPs based on their ability to crosslink to RNA.

Table S7. RBP potential for regulatory roles in virus-infected cells, related to Figure 7 List of RBPs reported in this study linked to the genome-wide screens that have reported their involvement in virus infection.

Table S8. Antibodies and oligonucleotide primers used in this study, related to STAR Methods

Table S9. List of GO terms and KEGG pathways related to immune response and known RNA-binding domains, related to STAR Methods

Document S2. Article plus supplemental information
