# Supplementary material for: Global analysis of protein-RNA interactions in SARS-CoV-2-infected cells reveals key regulators of infection
Source: Mol Cell. 2021 Jul 1;81(13):2851–2867.e7. doi: 10.1016/j.molcel.2021.05.023 (PMC8142890; doi:10.1016/j.molcel.2021.05.023)
Supplement: Document S1. Figures S1–S7 [file mmc1.pdf]

Molecular Cell, Volume *81*

Supplemental information

Global analysis of protein-RNA interactions in SARS-CoV-2-infected cells reveals key regulators of infection

Wael Kamel, Marko Noerenberg, Berati Cerikan, Honglin Chen, Aino I. Järvelin, Mohamed Kammoun, Jeffrey Y. Lee, Ni Shuai, Manuel Garcia-Moreno, Anna Andrejeva, Michael J. Deery, Natasha Johnson, Christopher J. Neufeldt, Mirko Cortese, Michael L. Knight, Kathryn S. Lilley, Javier Martinez, Ilan Davis, Ralf Bartenschlager, Shabaz Mohammed, and Alfredo Castello

Molecular Cell, Volume *81*

Supplemental information

Global analysis of protein-RNA interactions in SARS-CoV-2-infected cells reveals key regulators of infection

Wael Kamel, Marko Noerenberg, Berati Cerikan, Honglin Chen, Aino I. Järvelin, Mohamed Kammoun, Jeffrey Y. Lee, Ni Shuai, Manuel Garcia-Moreno, Anna Andrejeva, Michael J. Deery, Natasha Johnson, Christopher J. Neufeldt, Mirko Cortese, Michael L. Knight, Kathryn S. Lilley, Javier Martinez, Ilan Davis, Ralf Bartenschlager, Shabaz Mohammed, and Alfredo Castello

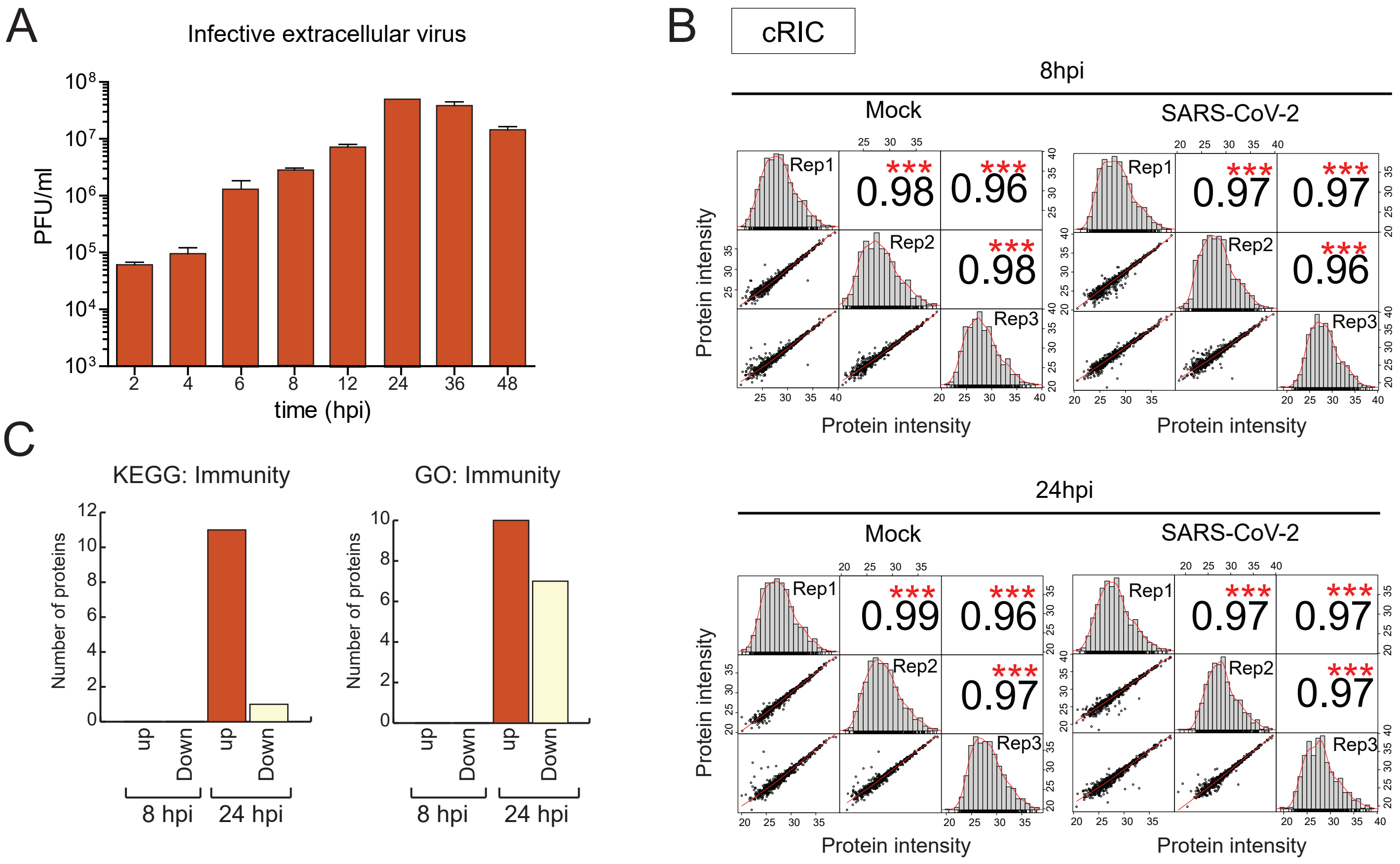

Figure S1: Profiling RBP dynamics by comparative RIC. Related to Figure 1. A) Supernatant of cells infected with SARS-CoV-2 for different times, were collected and titrated by plaque assay. B) Scatter plots comparing protein intensity [log2] across replicates of the total proteome analysis and the different conditions. Pearson correlation is indicated. ***, p<0.001. C) Number of upregulated or downregulated RBPs with annotation related to immunity in KEGG (left) or gene ontology (GO, right).

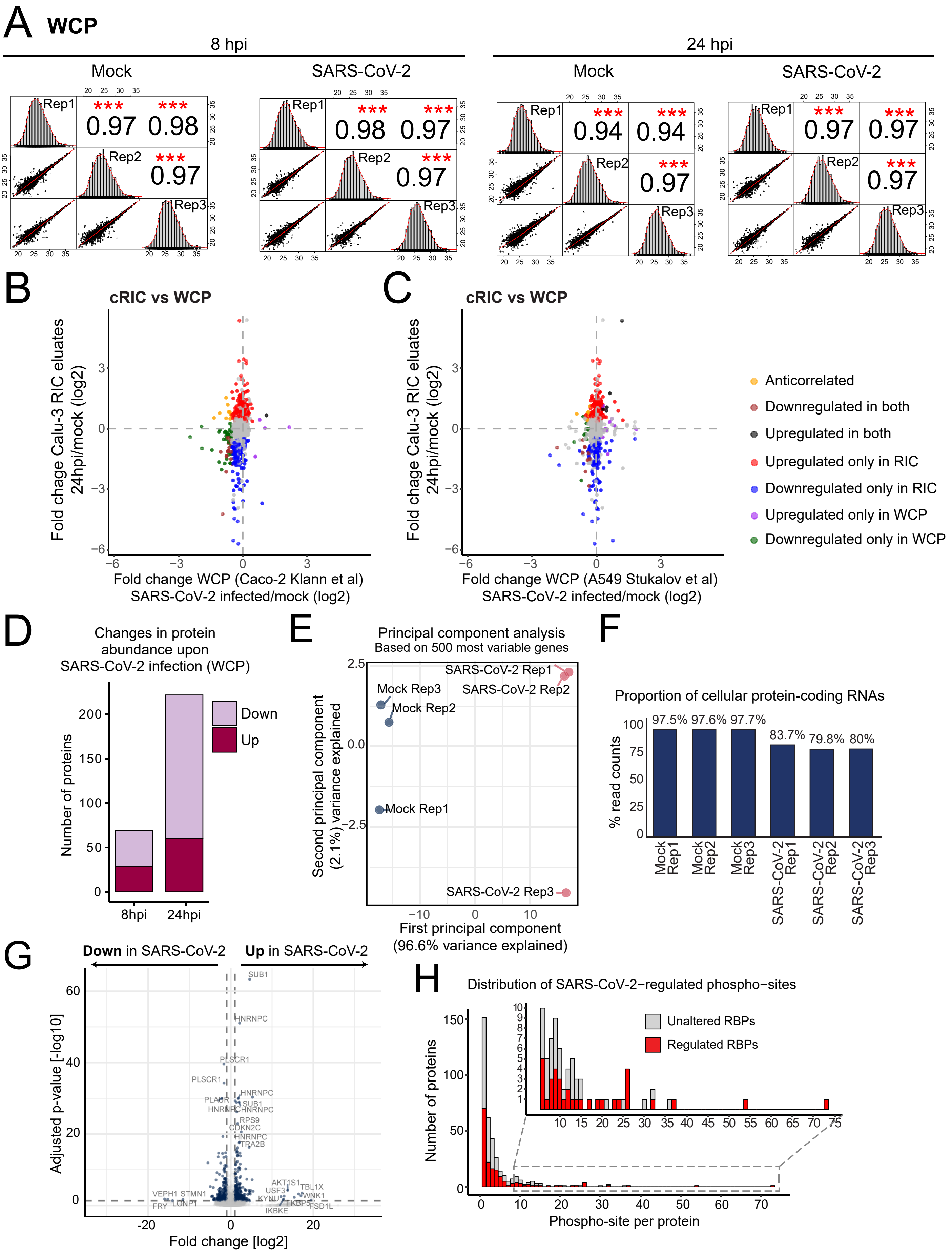

Figure S2: SARS-CoV-2 induced alterations in the whole cell proteome. Related to Figure 2. A) Scatter plots comparing protein intensity [log2] across different replicates. Pearson correlation is indicated. ***, p<0.001. B-C) Scatter plot showing the fold changes between 24 hpi and uninfected cells in our cRIC dataset (y axis) and the WCP (x axis) from Klann et al., 2020 (B) and Stukalov et al., 2020 (C). D) Bar-plot showing the proportion of proteins with changes in protein levels upon 8h or 24h of SARS-Cov-2 infection. E) First two components of a principal component analysis (PCA) performed on the 500 genes showing the highest variance in RNA-seq. The first component clearly separates infected cells from uninfected cells (mock in Blue and infected cells in Red). F) Percent of RNA-seq reads assigned to human protein coding genes of total count of uniquely assigned reads. G) Alternative splicing in SARS-CoV-2 infected cells. Volcano plot showing the fold change and adjusted p-value of the exon prevalence in mock and SARS-CoV-2 infected cells at 24hpi. H) Distribution of the number of phosphosites detected in regulated and unaltered RBPs.

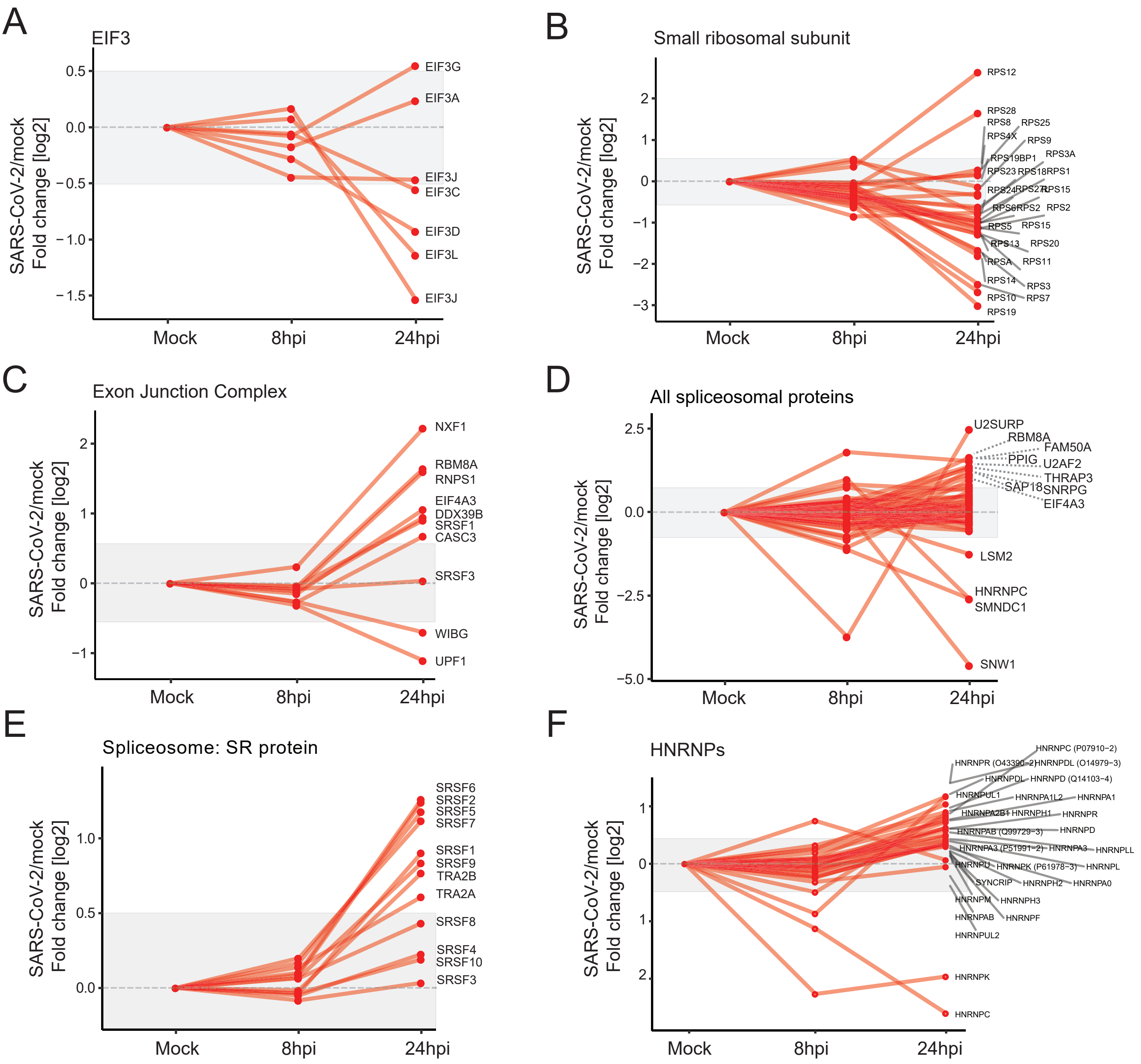

Figure S3. RNA-binding dynamics of functionally related RBPs in response to SARS-coV-2 infection. Related to Figure 3. A-F) Line plots showing the protein intensity ratio between 8hpi/mock and 24hpi/mock samples from the cRIC experiment for functionally related proteins, including EIF3 complex (A), small ribosomal subunit (B), exon junction complex (C), spliceosome (D), SR proteins (E)

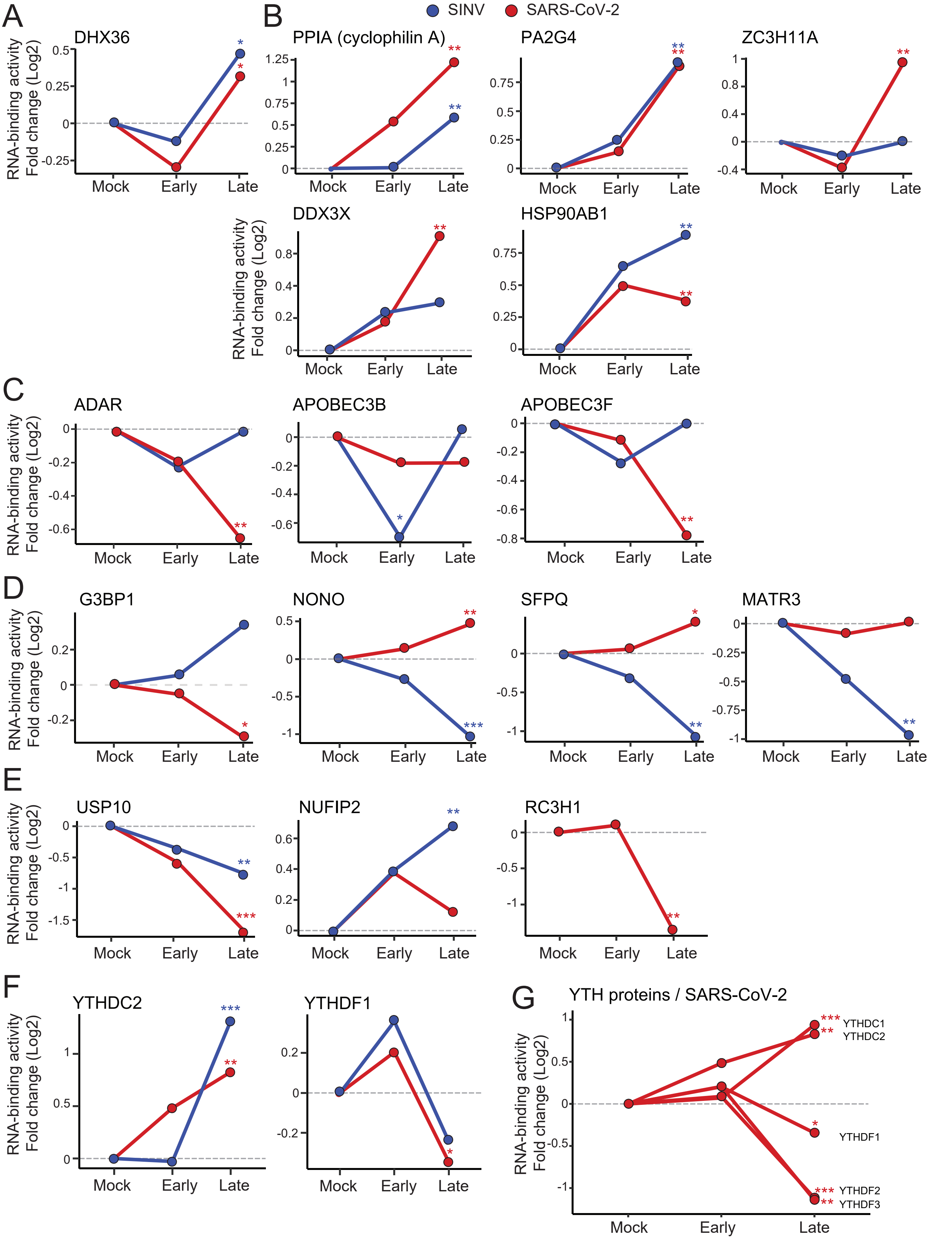

Figure S4. Comparison of the RBP responses to SARS-CoV-2 and SINV infection. Related to Figure 4. A-F) Line plots showing the protein intensity ratio between early/mock and late/mock samples from the SARS-coV-2 (red) and SINV (blue) cRIC experiment for selected proteins. Early was defined as 8 hpi for SARS-CoV-2 and 4hpi for SINV. Late was defined as 24 hpi for SARS-CoV-2 and 18 hpi for SINV. G) Line plot showing the protein intensity ratio between 8hpi/mock and 24hpi/mock for all the YTH m6A readers detected in the cRIC experiment *, FDR < 20% ; **, FDR < 10% and *** FDR < 1%.

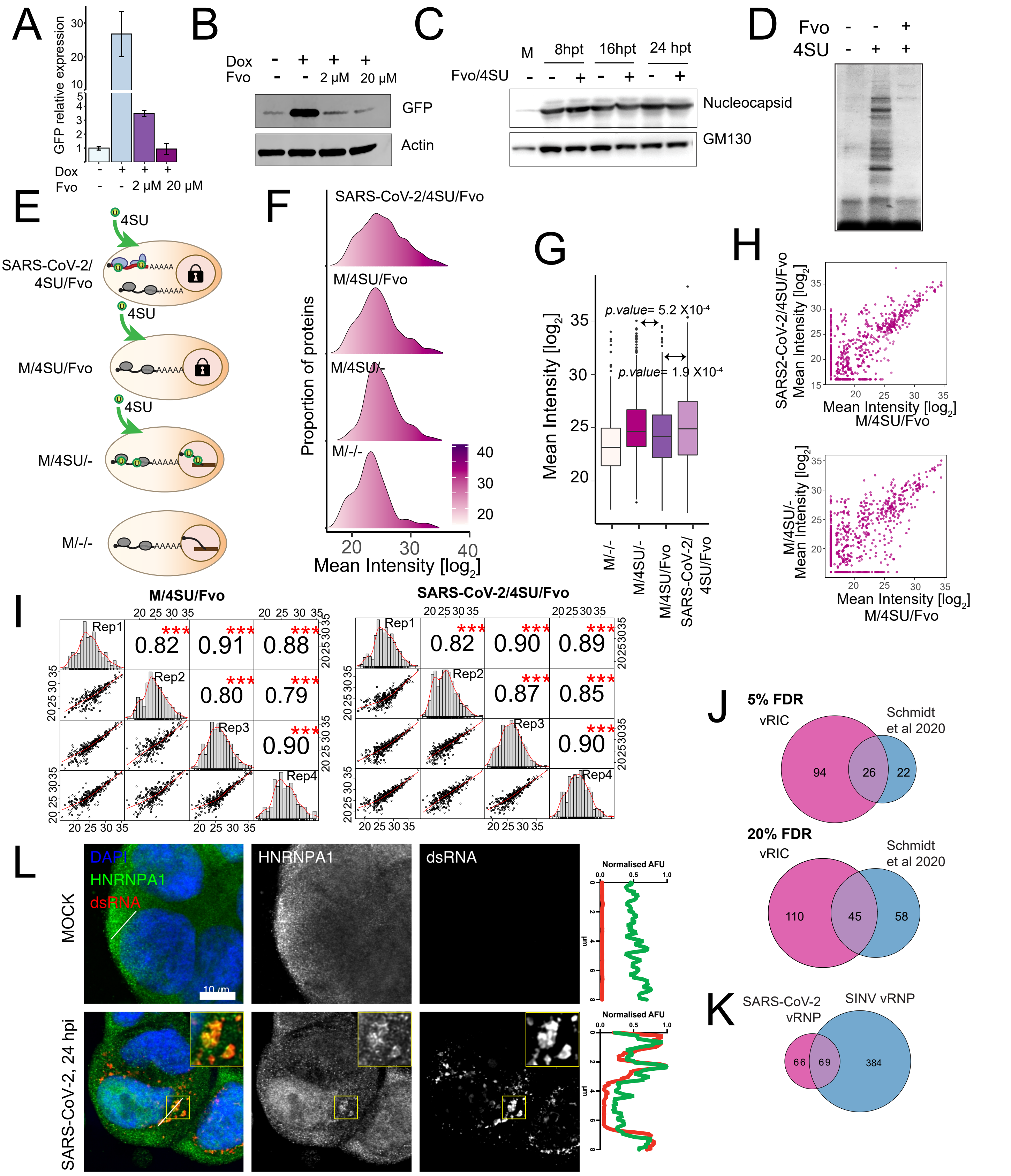

Figure S5. vRIC analysis of SARS-CoV-2 infected cells. A-B) Analysis of Fvo effects in transcription. Related to Figure 5. Flp-In T-REx HEK293 cells expressing eGFP under a tetracycline-regulated cytomegalovirus promoter were treated with doxycycline and different concentrations of Fvo. Expression of eGFP was assessed by RT-qPCR (A) and Western blotting (B). C) The effects of Fvo in SARS-CoV-2 infection were tested at different times post infection. Expression of NCAP was analysed by Western blotting. D) Silver staining analysis of the inhibitory effect of Fvo in the incorporation of 4SU into poly(A) RNA in Hek293 cells. Uninfected cells were treated with or without fvo and with or without 4SU and irradiated with 365 nm UV light. E) Schematic representation of all the controls used in the vRIC experiment. RBPs bound to poly(A) RNA were isolated by RIC and eluates analysed by silver staining. Fvo strongly reduces the purification of RBPs by oligo(dT) capture suggesting lack of incorporation of 4SU into nascent RNAs. F) Kernel density plot for the different vRIC samples showing the distribution of mean protein intensities. G) Box plot showing the protein intensity distribution in samples and controls of the vRIC experiment. P value is estimated by Welch's t-test. H) Correlation of protein intensity in the vRIC experiment when comparing infected vs uninfected cells and uninfected cells treated or not with Fvo. I) Scatter plots comparing protein intensity correlation between vRIC replicates for each condition. Pearson correlation is indicated. ***, p<0.001. J) Venn diagrams showing the overlapping between SARS-CoV-2 vRIC and the RAP-MS dataset generated by Schmidt et at 5% and 20% FDR. K) Venn diagram showing the overlapping between SARS-CoV-2 and SINV vRIC datasets. L) Immunofluorescence analysis using antibodies against HNRNPA1 and dsRNA in uninfected and infected cells. Right plot shows the distribution of fluorescence intensity in the green and red channels across the lines depicted in the image. AFU, arbitrary fluorescence units.

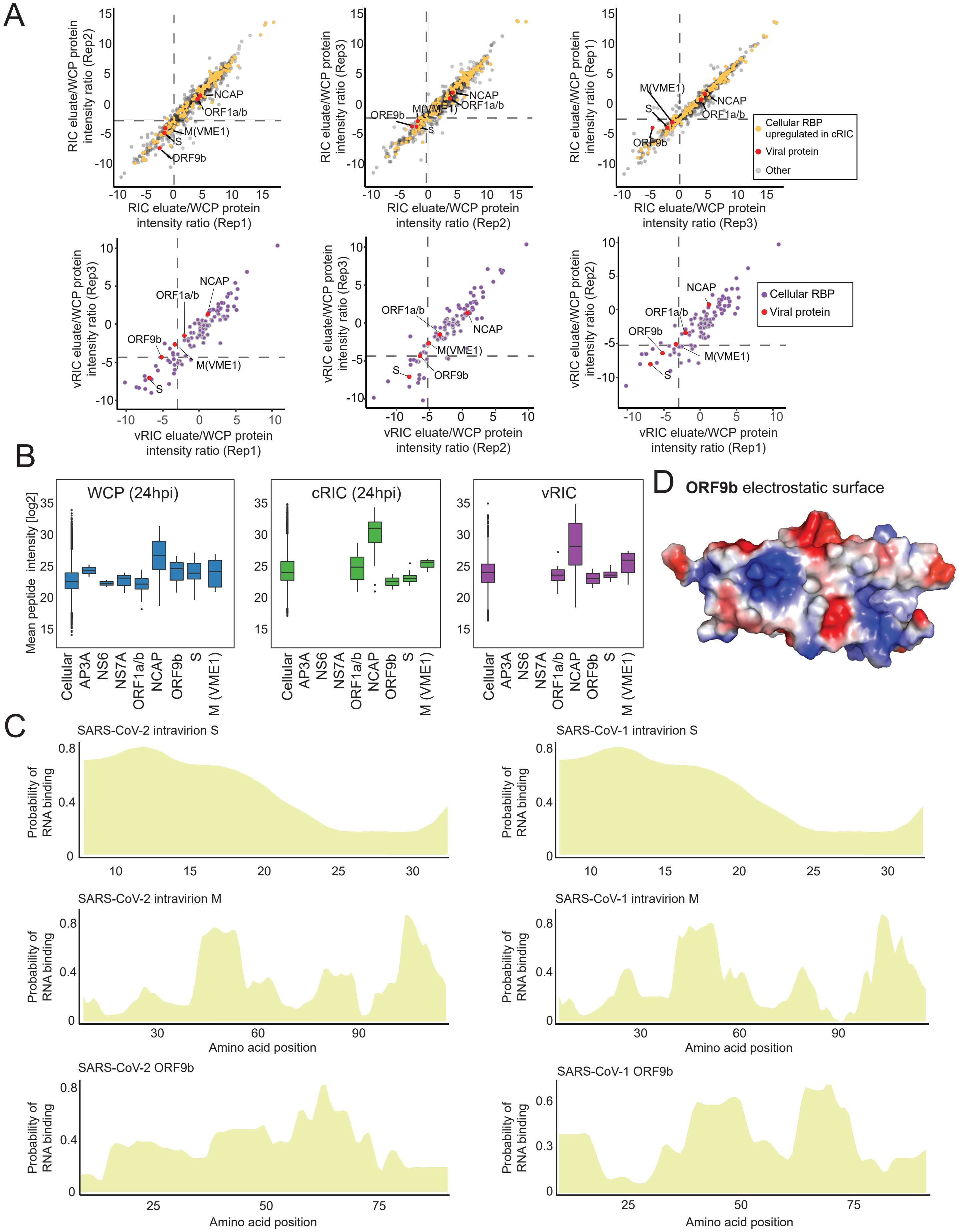

Figure S6. Analysis of SARS-CoV-2 proteins that interact with RNA. Related to Figure 6. A) Scatter plot showing the correlation between replicates of the protein intensity ratio between cRIC and WCP (upper panels) or vRIC and WCP (bottom panels). B) Peptide intensity distribution for all the viral proteins in WCP, cRIC or vRIC at 24hpi. C) Prediction of putative RNA-binding sites within the SARS-CoV-2 (left) and SARS-CoV-1 intravirion part of S (upper panels) and M (middle panels) or full length ORF9B (bottom panels). Prediction was made with RBDetect, which employs shrinkage discriminant analysis in the positive and negative examples within the RBDmap dataset to predict RNA-binding sites based on sequence similarities with human RBPs. D) Visualisation of the electrostatic surface of ORF9b using an available 3D structure (PDB ID: 6z4u). In blue are displayed the positively charged surfaces, while the negatively charged ones are shown in red.

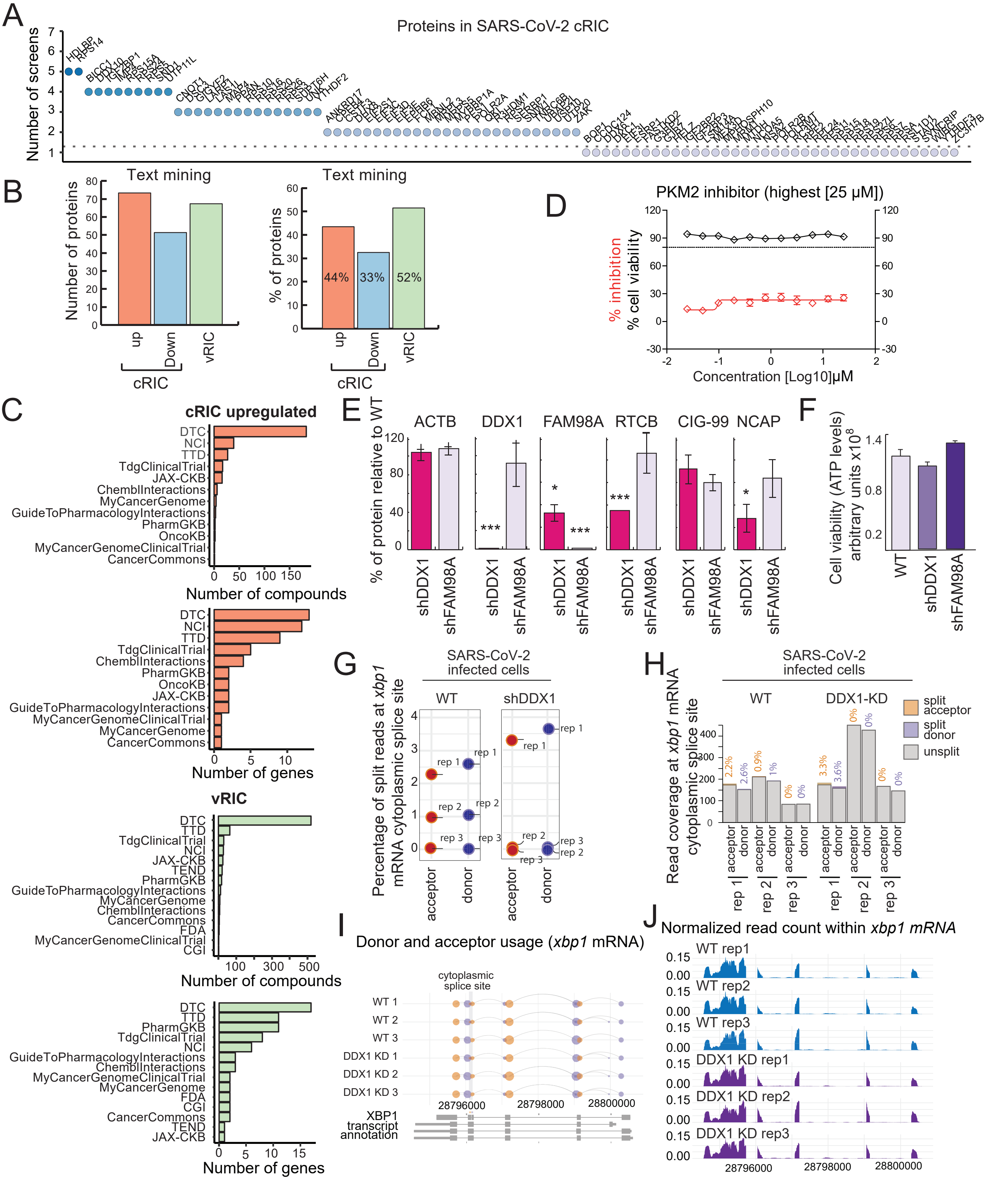

Figure S7. Functional implications of RBPs in SARS-CoV-2 infection. Related to Figure 7. A) Proteins with identified phenotypes in genome-wide screens using viruses. Proteins dowregulated in the cRIC experiment are displayed along the x axis, while y axis indicates the number of screens in which the protein has caused a phenotype in infection. B) Proportion of proteins within the cRIC and vRIC datasets that have been linked to infection using Pudmed automatized analysis. C) Comparison of RBPs upregulated by cRIC or/and present in the vRIC dataset to drug databases. D) Effect of PKM2 inhibitor on SARS-Cov-2 infection. Red line indicates the effects in infection measured by protein ELISA at each drug dose. Black line shows cell viability at each drug dose. Error bars are SEM from three independent experiments. E) Effects of DDX1 and FAM98A knock down in the tRNA-LC subunits and SARS-CoV-2 NCAP. Data is normalised to wild type cells. Error bars represent standard deviation using information from three biological replicates. *, p< 0.05; **, p<0.01 and *** p<0.001. F) Effects of DDX1 and FAM98A knock down on cell viability (cellular ATP levels). G) Percentage of split reads supporting cytoplasmic splicing of xbp1 mRNA in WT and shDDX1 cells infected with SARS-CoV-2. H) Read count at Xbp1 mRNA cytoplasmic splicing donor and acceptor versus reads mapping to the intronic region in WT and shDDX1 cells infected with SARS-CoV-2. While the overall counts are high, only a small percentage of reads support tRNA-LC mediated cytoplasmic splicing. I) Xbp1 mRNA splicing donor and acceptor usage according to split reads spanning each junction. J) Read coverage at Xbp1 mRNA exons and introns in SARS2-infected WT and DDX1 KD cells normalised to total reads mapped to Xbp1 mRNA. Xbp1 mRNA expression is similar in all samples.
